# Hybrid Polymer-Silica Nanostructured Materials for Environmental Remediation

**DOI:** 10.3390/molecules28135105

**Published:** 2023-06-29

**Authors:** Antonio Grisolia, Gianluca Dell’Olio, Angelica Spadafora, Marzia De Santo, Catia Morelli, Antonella Leggio, Luigi Pasqua

**Affiliations:** 1 Department of Environmental Engineering, University of Calabria, via P. Bucci, 87036 Arcavacata di Rende (CS), Italy; grisoliantonio@gmail.com (A.G.); giadellolio@gmail.com (G.D.); angelica.spadafora94@gmail.com (A.S.); 2 Department of Pharmacy, Health and Nutritional Sciences, University of Calabria, via P. Bucci, 87036 Arcavacata di Rende (CS), Italy; marzia.desanto@unical.it (M.D.S.); catia.morelli@unical.it (C.M.)

**Keywords:** environmental remediation, hybrid polymer-silica nanostructured materials, engineered nanostructured materials

## Abstract

Due to the ever-growing global population, it is necessary to develop highly effective processes that minimize the impact of human activities and consumption on the environment. The levels of organic and inorganic contaminants have rapidly increased in recent years, posing a threat to ecosystems. Removing these toxic pollutants from the environment is a challenging task that requires physical, chemical, and biological methods. An effective solution involves the use of novel engineered materials, such as silica-based nanostructured materials, which exhibit a high removal capacity for various pollutants. The starting materials are also thermally and mechanically stable, allowing for easy design and development at the nanoscale through versatile functionalization procedures, enabling their effective use in pollutant capture. However, improvements concerning mechanical properties or applicability for repeated cycles may be required to refine their structural features. This review focuses on hybrid/composite polymer-silica nanostructured materials. The state of the art in nanomaterial synthesis, different techniques of functionalization, and polymer grafting are described. Furthermore, it explores the application of polymer-modified nanostructured materials for the capture of heavy metals, dyes, hydrocarbons and petroleum derivatives, drugs, and other organic compounds. The paper concludes by offering recommendations for future research aimed at advancing the application of polymer-silica nanostructured materials in the efficiency of pollutant uptake.

## 1. Introduction

### 1.1. The Present State of Environmental Pollution 

The mitigation of environmental pollution and the reduction of polluting emissions are among the greatest challenges facing the world in the coming decades. This “environmental crisis” is predominantly attributed to the environmental and ecological changes resulting from the development processes of the “economic and technological man” of both the present and past centuries. Human activity is causing the deterioration of the environment through the depletion of both drinking water reserves and other environmental matrices. These resources face significant threats caused by various factors, primarily the pressures exerted by human activities (the estimated population will reach nine billion people by 2050) [1], increasing dependence on energy-consuming and ecologically damaging technologies and the expansion of industrial and agricultural activities [2]. Every day these factors contribute to the continuous release of huge quantities of pollutants into the environment originating from both organic and inorganic sources [3]. Another critical and imminent threat to the survival of our planet is the uncontrolled increase in greenhouse gas emissions, in particular CO_2_, NO_x_, and CH_4_, as well as the release of volatile organic pollutants (VOCs) and persistent organic pollutants (POPs) [4,5]. The practical steps to improve the current situation undoubtedly involve reducing pollutant emissions; however, the environmental matrices, which are already contaminated, require remediation operations.

### 1.2. Treatment Methods

In the past, numerous forms for the decontamination of environmental matrices have been proposed, using various existing chemical-physical techniques such as oxidation [6], ion exchange [7,8], reverse osmosis [9], adsorption [10,11,12,13,14] and others [15,16,17,18,19,20,21,22,23,24,25]. These techniques are widely used, although each of them has its own set of advantages and disadvantages [26].

Recently, numerous materials active in adsorption have been developed. These materials encompass classical adsorbents such as zeolites [27], alumina, activated carbon [28], biochar [29,30], bentonite [31], and others. An alternative efficient approach for remediation involves the application of silica-based nanostructured materials. Specifically, mesoporous silica-based materials allow extensive and suitable material engineering in bionanotechnology, nanomedicine, and analysis [32,33].

### 1.3. Silica Nanoparticles: Characteristics and Applications 

Silica nanoparticles present a stable structure [34], a high specific surface area, an adjustable pore structure, and a high density of silanol groups on their surface [35,36]. These characteristics enable easy modifications [37] and make them promising candidates as adsorbents for pollutants. Silica nanoparticles can be classified into various types based on their properties, synthetic approaches, and potential applications. Recent studies have revealed significant opportunities in the adsorption of a wide range of target substances, including metal ions, dyes, and other organics [38]. Moreover, the engineering of silica nanoparticles allows the development of tailored and selective scavengers to remove trace amounts of both organic and inorganic pollutants [39,40]. In such cases, UV-visible spectrophotometry is widely used as a detection technique due to its ease of use and ability to provide accurate results [41]. Moreover, because silica nanoparticles possess significant surface area, they can be suitably functionalized to selectively engage with particular pollutants present in a given environmental matrix. 

Most of the employed silica nanoparticles demonstrate remarkable versatility in complex matrices. They can effectively function even under harsh conditions: such as non-optimal pH levels or temperatures, as well as in the presence of high pollutant concentrations [42,43].

### 1.4. Hybrid Polymer—Silica Nanoparticles 

Although commonly functionalized nanoparticles exhibit valuable features, they cannot always ensure satisfactory adsorption performance. In such situations, it may be necessary to make additional adjustments to enhance the concentration of functional groups able to interact with pollutants. Polymers, therefore, can be used to improve the chemical and physical characteristics of these hybrid/nanocomposite materials [44,45]. 

The presence of multiple functional groups on the nanoparticles increases the capacity for complexing pollutants [46], furthermore, when a polymer shell is applied to the nanoparticle surface, it acts as a protective barrier against acidic or basic attacks from pollutants [38]. 

In addition to protection, polymers also allow extended contact time with the contaminated matrix and increase the colloidal stability of the nanoparticles [38].

Polymer-based nanocomposites often exhibit superior chemical, physical, and mechanical properties compared to unfunctionalized nanoparticles and isolated polymers. These versatile materials contain different functional organic and/or inorganic components making them highly effective for the removal of hazardous metal ions from wastewater [47]. 

This review focuses on hybrid/composite polymer-silica nanostructured materials used for adsorbing heavy metals, industrial dyes, and other organic pollutants from both aqueous and gaseous phases.

## 2. Synthesis and Functionalization of Mesoporous Silica

### 2.1. Synthesis of Silica Nanostructured Materials

Using nanostructured materials for developing innovative solutions for environmental cleanup generates considerable interest. In recent years, scientific research has increasingly focused on developing systems at the nanoscale with pores or channels classified as microporous, mesoporous, or macroporous as defined by the IUPAC (International Union of Pure and Applied Chemistry). Mesoporous materials are preferred over microporous and macroporous materials for a wide range of applications due to their larger surface area, adjustable internal and external surfaces, and accessible pore and channel sizes. These features enable efficient interaction with molecules present in the surrounding environment; additionally, mesoporous materials are biocompatible, thermodynamically stable, and cost-effective to be synthesized, making them a promising candidate in chemical research. Discussions are ongoing on aspects related to their synthesis and potential future applications. Among the several different kinds of nanostructured mesoporous inorganic materials, mesoporous silica nanoparticles have attracted considerable attention and interest [36,48]. 

Silica nanoparticles are composed of silicon and oxygen, generated through the hydrolysis and condensation of tetra-alkoxysilane Si(OR)_4_ groups. They are chemically defined as silicon dioxide (SiO_2_) and are characterized by the presence of surface hydroxyl groups, defined as “silanols” attached to the silicon atom. Their chemical structure is therefore composed of a three-dimensional network of amorphous silica, with a large specific surface area and a high density of silanol (-Si-OH) groups both on the external surface of the particle and the internal surface of the pores [49].

The first synthesis of mesoporous silica dates back to the 1990s when researchers from the Mobile Research and Development Corporation successfully produced mesoporous silica materials with ordered and well-defined pore structures. The synthesized materials, known as the M41S family, include MCM-41 a member characterized by hexagonal order. A template-based synthesis method was employed to achieve this level of organization in the inorganic walls. The process involved the poly-condensation hydrolysis of tetramethylortho or tetraethylortho silicate on the surface of surfactant micelles: this approach, known as the liquid crystal templating method, is based on dissolving a surfactant in water to create a foundation nucleus for silica structure formation.

Furthermore, it involves a sol-gel phase transition, wherein silica polymerization occurs around the surfactant micelles. Typically, this process takes place in the presence of an acid or basic catalyst and involves the hydrolysis and condensation of metal alkoxides (such as Si(OR)_4_) or inorganic salts in the sol phase, resulting in the formation of colloidal particles and a gel phase with a three-dimensional structure due to the cross-linking of siloxane bonds [50]. In general terms, the synthesis process of mesoporous silica materials involves the presence of a silica precursor, a surfactant that acts as a templating agent, and a catalyst. The synthetic process involves dissolving a templating agent in a solvent followed by the addition of the silica source while continuously stirring to facilitate hydrolysis and polycondensation. The process continues by raising the temperature to drive the condensation and eventually adding additives or changing the pH. The resulting products are then recovered, washed, and dried. The removal of the structuring agent takes place through solvent extraction or calcination. The description of the silica particle formation mechanism can be divided into three phases: nucleation, growth, and stabilization. In the initial phase of nucleation, silica nuclei are formed through the hydrolysis and condensation of inorganic precursors, such as tetraethyl orthosilicate (TEOS), in the presence of an organic templating agent. The growth phase is characterized by the progressive deposition of silica layers on the surface of the silica nuclei leading to the formation of an initial mesoporous structure. In a study conducted by Martin et al. it was observed that during the first 40 s of the hydrolysis of tetramethyl orthosilicate (TMOS), silicate ions adsorb around the surfactant micelles, gradually reducing their charge. This gradual adsorption process decreases the intermicellar repulsion resulting in the formation of small silica aggregates. After approximately 400 s, ordered silica mesopores with a hexagonal geometry were observed within the reaction mixture. Finally, through thermal treatment, such as calcination, the organic templating agent is removed resulting in the formation of a silica-porous structure [51]. 

Since the initial synthesis in the 1990s, several syntheses and methods have been developed leading to the discovery and production of new families of mesoporous materials. In 1998, Zhao’s research team at the University of California, Santa Barbara, successfully produced a porous material named SBA-15 (Santa Barbara Amorphous No. 15) by employing a three-block amphiphilic copolymer as a template. In comparison to MCM-41, the resulting SBA-15 material exhibited larger pores and thicker walls, arranged in a hexagonal order [52]. Over time, different kinds of mesoporous silica have been introduced using different structure directing agents (SDA) and synthesis conditions. In addition to MCM-41 and SBA-15, other well-known mesoporous silica materials include MSU (Michigan State University), FSM (Folded Sheet Materials), FDU (Fudan University), KIT (Korean Advanced Institute of Science and Technology) [53]. 

Each type of silica material is characterized by distinct structural and morphological properties that make them more or less suitable for specific practical applications. The morphology and size of the pores play a crucial role in affecting the properties of mesoporous silica-based nanoparticles. In a comparative study conducted by Gawande et al., the characteristics of MCM-41, SBA-15, and KCC-1 were investigated. Their findings revealed that the dendritic fibrous morphology of KCC-1 allows easier diffusion of reactive molecules into active sites compared to the tubular pores of MCM-41 and SBA-15 [54]. 

Within the open literature, many papers report innovative synthetic methods and approaches for producing silica-based nanoparticles showing improved properties in different fields such as catalysis, biomolecules immobilization, controlled drug release, as well as the removal of environmental pollutants, heavy metals and radioactive compounds from water, soil, and air. Nanoparticle pore size can be modified by carefully selecting surfactants and initial precursors offering precise control over the resulting structures. Inverse microemulsion, flame synthesis, and sol-gel process are the most commonly used methods for synthesizing silica nanoparticles. In the inverse microemulsion method, surfactant molecules and organic solvents combine to form spherical micelles. When water is present, the polar head group of the surfactant arranges itself to create microcavities that encapsulate water, commonly known as inverse micelles. However, the inverse microemulsion approach presents some drawbacks such as high costs and challenging removal of the surfactant from the final product [55]. Flame synthesis, on the other hand, is a prominent method for producing silica nanopowders by reacting silicon tetrachloride (SiCl_4_) with hydrogen and oxygen. However, the main disadvantage associated with flame synthesis is the difficulty in controlling the morphology and size of the resulting particles [56]. The sol-gel method involves the hydrolysis and condensation of monomers in a colloidal solution (sol) that serves as a precursor for the development of a structured network (gel). This method is extensively used due to its ability to regulate the size, distribution, and morphology of the resulting particles through precise control of reaction parameters [57].

In order to obtain well-defined and ordered mesoporous silica, careful planning of the production process is essential. This involves selecting an appropriate silica precursor and employing suitable compounds that can guide its structural development. Further investigations have revealed that by varying the surfactant, reaction conditions, and starting precursors it is possible to modify the diameter, shape, and connectivity of nanoparticle pores leading to significant structural changes in the final product [58]. Traditionally, the synthesis of mesoporous materials is based on the use of a cationic quaternary ammonium surfactant. In a study conducted by Wang et al., various ionic liquid surfactants, such as N-hexadecyl-N-methylpiperidinium bromide, N-hexadecyl-N-methylmorpholinium bromide or N-hexadecyl-N-methylpyrrolidinium bromide were investigated. The results of this study showed that the use of these surfactants led to the production of MCM-41 with a larger specific surface area and a more ordered pore structure compared to the originally synthesized MCM-41 [59]. Through a combination of surfactant swelling strategies, stirring and sonication techniques, Egger et al. obtained mesostructured silica nanoparticles characterized by high pore volumes. In their study, the authors used cetyltrimethylammonium bromide (CTAB) as the surfactant agent, dimethylhexadecylamine (DMHA) as the co-surfactant agent, and decane as the oil phase [60]. A straightforward method was employed to synthesize hollow mesoporous silica nanoparticles with a diameter of 25 nm in an alcohol-based chemical system using TEOS as the silica source and CTAB as the surfactant within a polystyrene template [61]. Bordoni et al. further demonstrated that the pore size of the synthesized material can be expanded through post-synthesis treatment involving the use of pore swelling agents and microwave irradiation. This approach was applied to non-calcined silica-based mesoporous materials and is compatible with both ionic and non-ionic surfactants (e.g., cetyltrimethylammonium bromide (CTAB) and polypropyleneglycol-based surfactants) [62].

Nonporous silica nanoparticles are synthesized in the absence of surfactant. A study conducted by Joni et al. introduced the direct mixing technique for precipitating silica precursor, using Na_2_SiO_3_ (Sodium silicate) and HCl as the precipitant agent, instead of the conventional method of adding HCl dropwise. The resulting nanoparticles, obtained through this easily and rapidly applicable method suitable for large-scale production, exhibit acceptable size and distribution [63].

The application of the ultrasound-assisted sol–gel method presents a simple, low-cost, and efficient alternative for producing spherical silica particles within a relatively short reaction time. By employing ultrasounds in chemical synthesis and modification processes researchers successfully synthesized monodisperse, uniformly shaped and size-controlled spherical silica particles [64]. Stopic et al. employed the ultrasonic spray pyrolysis (USP) method to obtain exceedingly fine spherical silica particles from a colloidal solution. They demonstrated that this synthetic approach enables precise control over the size and morphology of the particles by adjusting the concentration of the precursor colloidal solution [65].

Several economical and environmentally friendly methods have been developed for the synthesis of silica nanoparticles. One notable approach involves utilizing sugarcane bagasse as a source of silica to obtain nanoparticles. Recent studies have highlighted the potential of these nanoparticles for water pollution removal applications [66,67,68]. Furthermore, silica particles including MCM-41 [69], MCM-48 [70], SBA-15 [71], and SBA-16 [72] have been successfully synthesized through a bottom-up process using silicate extracted from rice husks, which are considered waste resources. 

Once the synthesis of silica nanoparticles is achieved, they can be functionalized with specific chemical groups to confer additional properties. These modifications enable selective binding with other molecules or the ability to detect specific targets, thereby enhancing their functionality.

### 2.2. Hybridization/Polymerization Techniques

Surface functionalization of silica-based materials is a rapidly expanding practice that enables the incorporation of appropriate and advanced features to nanostructures in order to facilitate their application in various scientific areas, particularly in the environmental field. This modification process takes advantage of the abundant and reactive silanol groups present on the nanomaterial surfaces, which serve as key sites for ligand anchoring [73]. By modifying the surface, properties such as hydrophilicity, hydrophobicity, acidity, basicity, chemical binding ability and stability can be greatly enhanced.

Surface modification of silica materials can be achieved through electrostatic interactions, or covalent conjugation, allowing the anchoring of various target compounds including polymers and other ligands onto the surface. There are two main strategies for surface functionalization of silica frameworks: post-synthesis grafting, where ligands are attached after the synthesis of the silica material and co-condensation, where the ligands are incorporated during the synthesis process itself [74]. 

In the co-condensation strategy, modifying agents and silica sources are added simultaneously or sequentially. Through hydrolysis, they condense around the structure directing micelles, resulting in the formation of functionalized nanomaterials. This one-pot synthetic method allows high-loading binding and facilitates the control of morphology [75,76]. Post-synthesis grafting approaches, on the other hand, involve the functionalization of active sites on the nanoparticle surfaces in suitable solvents used as reaction media, after the formation of the nanoparticle framework [77,78]. 

Hybrid materials have been obtained using a microwave-assisted grafting technique to introduce organic functional groups onto mesoporous silica, specifically of the MCM-41 type. This grafting procedure has proven to be highly effective for preparing hybrid organosilica in a solvent-free environment. The use of microwaves allows rapid and clean functionalization of mesoporous materials, and this method has been applied to produce a wide range of functionalized materials [79,80].

Hybrid mesoporous silicas have demonstrated their potential as matrices for enzyme immobilization. SBA-15 mesoporous silica particles functionalized with N-(2-Aminoethyl)-3-aminopropyl groups with a 4.4 nm pore diameter, have been used for trypsin immobilization providing an exceptional enzymatic bioreactor for protein digestion. The application of this system allowed the rapid generation of proteolytic fragments of myoglobin in as little as one minute after adding the protein to the trypsin-immobilized mesoporous support providing 100% sequence coverage. The presence of N-(2-aminoethyl)-3-aminopropyl and aminopropyl (AAPTES and APTES, respectively) in the functionalized SBA-15 materials demonstrated their potential in the field of digestion techniques for mass spectrometry-based proteomics [81].

The choice of solvent, in hybridization reactions with solvent, plays a crucial role in controlling the distribution of functional groups on the nanoparticle’s surface. It has been extensively documented that the use of polar-protic solvents leads to a lower accumulation of polar organic functional groups and yields nanoparticles with higher surface area. Conversely, dipolar-aprotic, and non-polar solvents promote a higher concentration of organic groups on the nanoparticle surface. Interestingly, when non-polar organosilanes were employed, a significantly lower degree of functionalization was observed with both polar-protic and dipolar-aprotic solvents [82].

Furthermore, the presence of water in the reaction medium plays a crucial role in the silica surface functionalization process. Gartmann et al. investigated the influence of water in the functionalization of MCM-41 type nanoparticles with APTES (3-aminopropyltriethoxysilane) in toluene with varying water content. Increasing the content of water, promoted the clustering of APTES molecules, leading to partial blocking of the pores and hindering the binding of additional alkoxylane molecules. Consequently, this resulted in a non-homogeneous surface modification and a poor functionalization of the pore structure [83].

The functionalization of silica nanoparticles with polymers has gained significant scientific interest in several, particularly in environmental remediation applications for air and water as well as in the adsorption of dyes. This is primarily due to the unique and improved characteristics of the modified material. Boukoussa et al. conducted a study aiming to improve the affinity of SBA-15 mesoporous silica for acidic CO_2_ molecules by the incorporation of base-like chemical species on its surface. This was achieved by in-situ polymerization of aniline within SBA-15 using different amounts of aniline, resulting in the formation of polyaniline/SBA-15 (pani/SBA-15) nanocomposites. The researchers investigated the surface basicity and hydrophilic character of the nanocomposites through the thermal programmed desorption of CO_2_ and water. These assessments revealed that the CO_2_ retention capacity significantly increased with higher polyaniline content compared to SBA-15 materials [84].

In a recent study conducted by Alswielesh, the surface of mesoporous silica nanoparticles (MSNs) was coated with zwitterionic polymer brushes derived from poly(2-(tert-butylamino)ethylmethacrylate) to increase the adsorption capacity of MSNs for rhodamine B and crystal violet in wastewater. The resulting polymer hybrid nanoadsorbent, named PTBAEMA-SO_3_H@MSNPs, consists of APTES functionalized MSNs on both the inner and outer surfaces, with PTBAEMA brushes uniformly grown. The sulfonic acid groups on polymeric chains were introduced through the oxidation of the thiol group anchored on PTBAEMA segments [85].

Polymer-based silica nanocomposites have attracted significant attention in various biotechnological fields due to their improved and even novel physical properties, including mechanical and thermal properties when compared to the starting materials. These composites offer a combination of the desirable characteristics of both inorganic materials, such as rigidity and thermal stability, and organic polymers, such as flexibility, ductility, processability, and dielectric properties. Moreover, the functionalization of silica with polymers allows for the enhancement of surface hydrophobicity [86]. Loy and co-workers achieved the production of stronger, low-density nanocomposite aerogels by using chemical vapour deposition and polymerization of cyanoacrylate onto aminated silica aerogels. This modification resulted in higher flexural strength compared to starting aerogels, while only increasing the density by a factor of 2.5. Furthermore, the modified aerogels were easily cut into desired shapes, providing added versatility in their applications [87]. The selective incorporation of viscoelastic poly(dimethylsiloxane) in graphene aerogels resulted in a synergistic nanocomposite with excellent combined mechanical and electrical properties. The nanocomposite exhibited fully reversible structural deformations and enhanced mechanical strength even after repeated compressive cycles. This structural reinforcement increased Si-O-Si connections between particles, thereby improving the mechanical properties of silica-based aerogels [88]. In the existing literature, a wide range of polymer modifications have been proposed based on the nature of polymers, the polymerization process, and the desired functionalization for specific applications. The incorporation of polymers in nanomaterials provides active sites for interactions that are strictly dependent on the types of polymers used. Polar active sites, such as hydroxyl, amines, and carboxylic groups are particularly relevant for interactions with organic polar molecules or metal ions, while non-polar carbon-chain sites are useful for non-polar compound interactions. These functional groups confer reactivity, stability, and solubility, which are crucial for several applications involving hybrid nanostructures in environmental restoration. However, the choice of starting monomer significantly affects the material characteristics and the resulting polymer network [89]. The synthesis of polymer can be adjusted to tailor porosity and the presence of active adsorption sites on their surfaces. This allows for the incorporation of various groups into the main or side chains of the polymers, including coordination groups, ionizable groups or permanent charged groups. These functional polymers can be used for the creation of polymer-based nanocomposites that are able to remove hazardous inorganic pollutants from water sources [90]. Moreover, the sorption properties of functional polymers, such as the sorption kinetics, can be finely tuned by considering changes in architecture, cross-linking density, and solubility. Insoluble polymeric sorbents are generally suitable for simultaneous sorption and separation processes. Additionally, for fast sorption kinetics, hydrosoluble polymers are required [91]. The most straightforward technique for preparing polymer-functionalized nanomaterials involves direct mixing, which can be achieved through melt blending and solution blending methods. According to the first approach, liquid-state polymers are mixed with silica nanoparticles at a temperature above their melting point or glass transition temperature (Tg). Alternatively, solution blending entails mixing nanoparticles and polymer in a solvent that is subsequently removed during the processing state [92]. Post-synthesis methods can be classified as general polymerization and surface-initiated polymerization. General polymerization involves mixing modified surface nanoparticles with polymers, in a solution or liquid form, leading to the formation of polymeric chains within the pores of the nanomaterial. The preliminary functionalization of nanoparticles allows enhanced interactions with monomers [93]. Surface-initiated polymerization involves the grafting of polymer chains onto the silica surface and may occur through two alternative routes: “grafting-to” and “grafting from”. In the first method, monomers with non-expanded chains anchored on nanoparticle surfaces, inhibit the grafting of other monomers and reduce the functionalization rate and density. In contrast, the “grafting-from” approach enables the growth of polymer chains directly on the silica surface, allowing for a high graft density [94].

Grafting density and graft length are two important parameters that need to be considered and optimized to confer suitable performances and properties to the processed polymers. The grafting-to technique has the advantage of characterizing the grafted length of polymers before their attachment. This approach is commonly employed with pre-formed polymers, that are easy to synthesize or commercially available end-functionalized polymers. However, it requires great effort in purification operations. The “Grafting from” process is selected when precise control over grafting density, graft length and polydispersity is desired. This technique is predominantly carried out in water, methanol, DMF, and DMSO, and has experienced significant advancements over the past decade [95]. Various categories of surface-initiated polymerization through the “grafting from” technique have been identified, such as free radical polymerization, ring-opening polymerization (ROP) and controlled radical polymerization (CRP), also known as surface-initiated reversible-deactivation radical polymerization (SI-RDRP). The latter technique includes surface-initiated atom transfer radical polymerization (SI-ATRP), surface-initiated reversible addition−fragmentation chain transfer (SI-RAFT) polymerization, surface-initiated nitroxide-mediated polymerization (SI-NMP), and surface-initiated photoiniferter mediated polymerization (SI-PIMP) [96]. One of the extensively investigated controlled radical polymerization methods is reversible addition-fragmentation chain transfer (RAFT) polymerization. Compared to the traditional SI-RAFT polymerization, there has been a significant interest in the visible light-regulated photo-inducted electron/energy transfer (PET)-RAF polymerization. Nechani and Joshi successfully produced mesoporous silica nanoparticles functionalized with a RAFT agent using an alternative stepwise approach, employing Eosin Y as a photo-catalyst. In comparison to conventional RAFT, the PET-RAFT polymerization technique offers several advantages, including reduced reaction time, simplified procedure requirements, as well as higher polymer functionalization. These hybrid mesoporous silica nanoparticles have emerged as more suitable nanomaterials with a wide range of potential applications, including nanoremediation [97]. More recently, Zheng developed a novel, selective and recyclable magnetic separation adsorbent, polymer-grafted silica-coated acid-resistant magnetic chitosan composite (PMSC) for the recovery of Pb (II) from aqueous solutions. Magnetic chitosan microparticles Fe_3_O_4_@SiO_2_@Chitosan (MSC) with core-shell structure, were coated through a free polymerization reaction using acrylic acid (AA) and sodium allylsulfonate (SAS) as grafting anionic monomers and potassium persulfate as initiator. The composite showed pH-responsive adsorption, which was six times higher compared to MSC, and it reached a maximum adsorption capacity of 88.03 mg/g at pH = 6. However, after five cycles of repeated use, the adsorption capacity decreased by approximately 20% compared to its initial value. The synthesized adsorbent composite demonstrated remarkable selectivity for Pb (II) when tested in a solution containing multiple metal ions [98].

## 3. Adsorption Applications

### 3.1. Adsorption of Heavy Metals Pollutants

**Figure 1:**
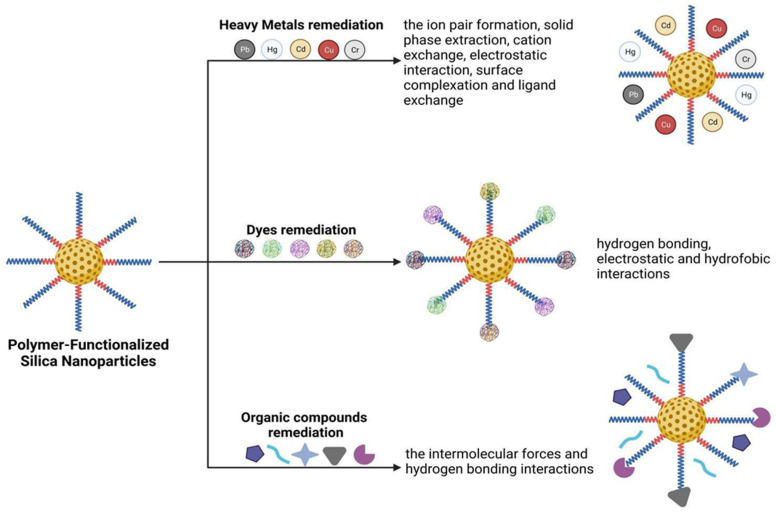
Applications of hybrid/polymers nanoparticles from reference [12].

The contamination of the ecosystem with toxic heavy metals, which are the major inorganic pollutants, has become an environmental problem that affects public health. Although these metals can also be released from natural sources such as volcanic activities, hot springs, erosion, etc., the majority of them derive from anthropogenic activities, particularly associated with urban expansion, industrial development and intensive agriculture. Activities such as the combustion of fossil fuels, vehicle emissions, mining, solid/liquid waste incineration, and industrial wastewater are the main sources of these pollutants. Consequently, heavy metals mainly contaminate natural water bodies and soils, as even those released into the atmosphere eventually settled on land. The most significant danger is that these persistent pollutants accumulate in the environment and gradually contaminate the food chains. When they enter the plant system through various physiological processes, they can be consumed by animals or humans through the consumption of vegetables grown on contaminated lands, or worse, through the consumption of water that has passed through such lands, that serves as the main pathway for heavy metal entry into the animal kingdom. Figure 1 provides a schematic representation of hybrid/polymers nanoparticles applications.

These heavy metals can accumulate in various food sources, including cereals, meat, fruit, fish, and molluscs. Moreover, their bioaccumulative nature poses a toxic threat to organisms even at extremely low concentrations [99,100,101]. Due to these reasons, the identification and removal of hazardous metals from the environment have become a fundamental challenge to ensure the efficacy of remediation procedures, in order to comply with concentration limits set by international standards. Nanotechnologies, employing nanoscale manipulation and size control, offer promising solutions to this environmental issue, by improving the quality of air, water, and soil.

**Figure 2:**
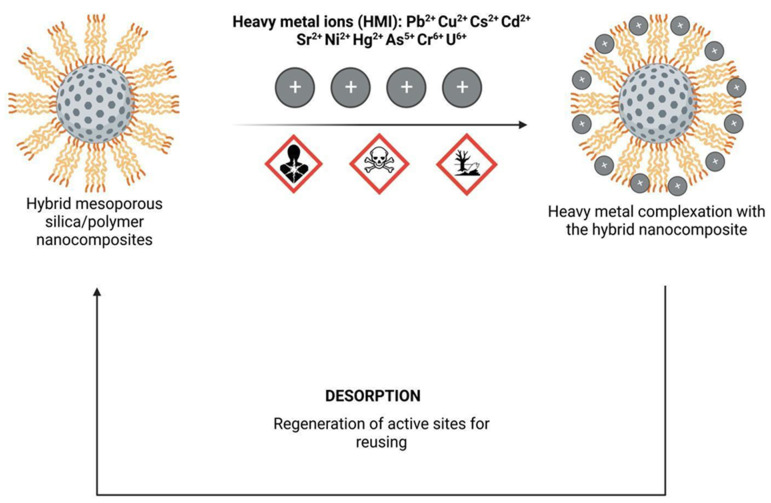
Hybrid polymer-silica applications in heavy metals complexation [38].

Most adsorbents based on mesoporous silica nanoparticles (MSN) focus on the removal of heavy metal ions (Pb, Cd, Cu, Cr, Zn, Ni, Hg, etc.) from water. The mesoporous structure of MSNs increases their surface area, thereby enhancing the availability of functional groups to adsorb metal ions. Hence, MSNs hold great potential as metal adsorbents, especially when functionalized with -NH_2_, -COOH, -SH groups as they further enhance their efficacy in complexing metal ions. Additionally, combining these nanomaterials with polymers that possess these groups, improves the chemical/physical properties of the polymers themselves, thereby boosting their efficiency in removing toxic chemicals [38,102,103]. A simplified illustration of heavy metal adsorption by hybrid polymer-silica is illustrated in Figure 2.

Mohammadnezhad et al. synthesized hybrid-silica MCM-41 and poly-methyl methacrylate (PMMA) nanoparticles using both in-situ polymerization and ultrasound irradiation techniques. These particles were then used for the adsorption of copper metal ions (Cu^2+^) ions from aqueous media, analysing the effect of pH, contact time, and initial concentration of the metallic ions. The adsorption performance was studied using two common isothermic models, Langmuir and Freundlich. The maximum value of adsorption capacity for Cu (II) ions was found to be 41.5 mg/g at optimal pH = 4, temperature of 25 °C, contact time of 140 min, and using 10 mg of adsorbent [104]. Even Plohl et al. developed an excellent, environmentally friendly, highly efficient nanobiosorbent for chelating Cu^2+^. This nanobiosorbent consists of a magnetic maghemite core of 13 nm, coated with a silica layer of approximately 7 nm forming a core-shell structure. The silica layer provides abundant reactive functional nucleophilic -OH groups. These reactive sites on the surface of silica were coupled with the amino-biopolymer carboxymethyl chitosan (CMC) through a reaction with carbodiimide in an aqueous environment, resulting in the formation of a strong and highly stable ester bond with the biopolymer.

The resulting nanoadsorbent exhibited superior copper adsorption capabilities compared to the ones developed by Mohammadnezhad, with an equilibrium capacity of 200 mg/g after a 2-h process and a maximum adsorption capacity of approximately 350 mg/g compared to other reported magnetic chitosan-based adsorbents. Adsorption models revealed the mechanism of spontaneous physical adsorption of Cu^2+^ on the heterogeneous surface of the nanoadsorbent. These environmentally friendly silica-coated MNPs based on amino-biopolymers represent an efficient, stable, and novel adsorbent for commercial applications in the removal of heavy metals. Furthermore, their reusability makes the proposed environmental technology economically viable and aligns with the concept of zero waste [105].

In 2022, Abass et al. utilized gamma irradiation to initiate a polymerization reaction of polyacrylonitrile-acrylamide, resulting in the synthesis of a novel silica-polymer hybrid nanocomposite known as P(AN-AM)-NS. This synthesized material was employed for the batch absorption of various metal ions, including copper (Cu^2+^), lead (Pb^2+^), cesium (Cs^+^), strontium (Sr^2+^), and cadmium (Cd^2+^) ions, from liquid media. The study examined the effects of agitation time, reaction time, and pH. The distribution coefficients indicated a selectivity order of Pb^2+^ > Cs^+^ > Cu^2+^ > Cd^2+^ > Sr^2+^ at the optimal pH = 4.3. The absorption capacity of the nanocomposite decreased as the temperature of the solid powder increased. The thermodynamic parameters showed an endothermic and spontaneous trend. Overall, the investigation demonstrated that the P(AN-AM)-NS nanocomposite is a suitable organic-inorganic sorbent for efficiently absorbing the studied ions from liquid solutions. This nanocomposite shows potential as a material for purifying effluents contaminated with these ions [106]. In 2020, Betiha et al. developed a mesoporous silica-polymer hybrid as an adsorbent for bivalent heavy metals, specifically Pb (II), nickel (Ni (II)), and Cu (II) using rice straw and polyvinylpyrrolidone (PVP) in three successive steps. The last step involved the formation of a Schiff base (PVP-SBA-15) between the amine-coated silica and PVP. The grafting of the PVP polymer introduces a high affinity towards heavy metal ions. The adsorption capacity of the hybrid material was evaluated through a series of experiments conducted using the Langmuir model. The results showed an adsorption capacity of 128 mg/g for Cu (II), 175 mg/g for Pb (II), and 72 mg/g for Ni (II) within a short timeframe of 30–60 min, and at a pH = 5. Moreover, this hybrid material demonstrated excellent performance for the adsorption of heavy metal ions from a wastewater sample, resulting in a reduction of 92% [107]. Recently, Xing et al. developed a hybrid system for the adsorption of Ni (II) ions by copolymerizing 1-vinyl-imidazole and acrylic acid on the surface of mesoporous silica microspheres. This was achieved through grafting polymer brushes using reversible-addition fragmentation chain transfer (RAFT) polymerization. The resulting materials (SiO_2_@poly(VI-co-AA)) were then used for the removal of Ni (II) from an aqueous solution, exhibiting a rapid adsorption rate. The study revealed that the optimal pH for adsorption was pH = 7, and the maximum adsorption capacity was determined to be 62.81 mg/g according to the Langmuir equation at a temperature of 298.15 K. The adsorption process reached equilibrium within 60 min, indicating a relatively fast adsorption rate. Moreover, the thermodynamic analysis revealed the spontaneous and exothermic nature of the adsorption process. Ni (II) ions could be eluted from the adsorbent using a 1 mol/L HCl solution, achieving a desorption efficiency of over 99%. The adsorbent also exhibited good reusability. Specifically, at an adsorbent dose of 6 g/L, the removal rate of Ni (II) from a solution with an initial concentration of 30 mg/L reached 99.40%, resulting in a residual Ni (II) concentration of 0.17 mg/L. Overall, these findings suggest that SiO_2_@poly(VI-co-AA) microspheres have the potential as adsorbents for the removal of nickel ions from wastewater and could be further explored for the removal of other heavy metals and dyes [108].

Hashami et al. synthesized a polymer using the surface imprinting technique. They used methacrylic acid on a mesoporous silica structure with a magnetic core composed of Fe_3_O_4_. Additionally, the structure was functionalized with -NH_2_ groups, resulting in the formation of Fe_3_O_4_@SBA-15-NH_2_-IIP. The inclusion of silicate structures in this polymer accelerated surface adsorption and facilitated the access of analytes to specific polymer cavities. The developed magnetic imprinted polymer SBA-15-NH_2_@Dual-Template was applied for solid-phase extraction and the determination of Pb^2+^ and Cd^2+^. The concentration range for Pb^2+^ was found to be 0.5–950 μg/L, with a correlation coefficient of 0.9988, a detection limit of 0.35 mg/L, and a relative standard deviation of 3.5%. For Cd^2+^, the corresponding values were 0.3–980 μg/L, 0.9969, 0.15 mg/L, and 2.4%, respectively. In conclusion, Fe_3_O_4_@SBA-15-NH2-IIP demonstrated the ability to adsorb and separate lead and cadmium simultaneously, exhibiting excellent selectivity (Q = 10.38 for Cd^2+^ and Q = 10.20 for Pb^2+^). Finally, the proposed method was successfully applied to various vegetable samples [109]. 

Zendehdel et al. investigated the adsorption of Pb (II) and Cd (II) from aqueous solutions, with the use of a series of hybrid nanocomposites, including polyacrylamide-co-acrylic acid/MCM-41. The results revealed that this nanocomposite exhibited higher adsorption capacity compared to the starting MCM-41 silica material. Specifically, the nanocomposite demonstrated a Pb (II) adsorption ranging from 90% to 99%, while Cd (II) adsorption was approximately 88% to 98% at room temperature. Adsorption increased up to 120 min for Pb (II) and 60 min for Cd (II) and occurred via an endothermic process. Moreover, a desorption study revealed that the nanocomposite maintained good adsorption behaviour for the metallic ions even after 10 cycles [110]. Soltani et al. have developed a novel nanocomposite material, named M-MCM-41/PVOH NC, specifically designed for the adsorption of Cd (II) from aqueous solutions. It can be considered “green” due to its composition, which incorporates an inexpensive and eco-friendly polymer known as poly(vinyl-alcohol) (PVA). The preparation of the nanocomposite involved a simple ultrasound-assisted mixing procedure incorporating the PVA polymer. The kinetic analysis revealed that the adsorption process of Cd (II) onto M-MCM-41/PVOH NC material followed the PSO kinetic model, representing chemical adsorption occurring primarily on the outer hydroxyl groups of PVA. To assess the adsorption equilibrium, various isotherms including Langmuir, Freundlich, and Dubinin Radushkevich equations were employed. The maximum adsorption capacity of M-MCM-41/PVOH NC for Cd (II) was determined to be 46.73 mg/g at 298 K on a typical monolayer saturated with a fixed number of localized adsorption sites. Furthermore, the nanocomposite was tested under diverse conditions, revealing a superior adsorption capacity for Cd (II) when compared to other silica-based composites. Overall, this newly developed material demonstrates promising characteristics as both an effective and environmentally friendly approach for removing Cd (II) from water [111].

Recognizing chitosan as one of the most commonly used biopolymers for the formation of polymer/silica nanocomposites, El Kurdi et al. developed a hybrid porous gel by combining silica nanoparticles with chitosan oligosaccharide lactate (COL). This hybrid gel, known as COL-Si, exhibits several advantageous properties, including a highly developed surface area, high thermal and acid stability, low cost, and high resistance to microbial degradation.

Notably, the COL-Si hybrid gel demonstrated remarkable selectivity for the adsorption of mercury ions (Hg^2+^), with the highest adsorption efficiency observed at pH = 8 and 9. Furthermore, the hybrid gel can also be used for the detection of Hg^2+^ using curcumin as a chromophore trapped within the gel. This detection method exhibited a very low detection limit of 10 ppm. Therefore, the COL-Si gel can be used as an effective adsorbent for mercury ions in wastewater treatment, and in combination with curcumin, can be used as a detector of Hg^2+^ in aqueous environments [103]. In the same year, Jumah et al. synthesized a new adsorbent called β-CD/MCM by combining the β-cyclodextrin polymer with mesoporous silica MCM-48. The objective was to remove arsenic (As (V)) and mercury (Hg (II)) ions from aqueous solutions using batch and fixed-bed column experiments. The adsorbent demonstrated a high adsorption capacity for both ions, even in the presence of anions or metal ions. Specifically, the maximum adsorption capacity for As (V) and Hg (II) was found to be 265.6 mg/g and 207.9 mg/g, respectively, superior to other adsorbents. Thermodynamic studies further revealed that the adsorption process was favourable, spontaneous, and exothermic. By employing β-CD/MCM as an adsorbent in a fixed-bed column system, a decontamination percentage of 72.8% for As (V) and 60.4% for Hg (II) was achieved in 8.1 litres of contaminated water. These results were obtained under specific operating conditions, including 5 cm bed thickness, 5 mL/min flow rate, 25 mg/L concentration, and pH6. In conclusion, the β-CD/MCM adsorbent exhibits promising adsorption properties for the removal of As (V) and Hg (II) ions in both batch and fixed-bed column studies [112]. In 2019, Yong Fu et al. developed a mesoporous silica nanocomposite with a magnetic Fe_3_O_4_ core. The outer surface of the nanocomposite was functionalized with poly(m-aminothiophenol) to enable the selective removal of Hg (II) ions from aqueous solutions. The experimental results revealed that the mesoporous silica/organic polymers nanocomposite (MMSP) exhibited a remarkable adsorption capacity, reaching 243.83 mg/g, and achieved a removal percentage of 97.53% within only 10 min at pH = 4. The presence of coexisting ions in the aqueous solutions did not significantly affect the selective removal of Hg (II) ions by the MMSP nanocomposite. Furthermore, the recovered adsorbent exhibited good adsorption performance even after five extraction cycles. Additionally, the waste adsorbent containing a considerable amount of Hg (II) was converted into a highly active catalyst, thereby reducing the economic and environmental impact associated with conventional adsorption methods. This catalyst proved to be efficient in the transformation of phenylacetylene to acetophenone with a yield of 97.06% [113].

Interesting applications of polymer-silica nanocomposites concern the capture of highly dangerous substances such as uranium. Amidoxime, an excellent amphoteric ligand with both acidic and basic sites in its structure, offers fascinating possibilities. It forms a stable five-membered chelate with uranium metal ions (U (VI)) by utilizing the lone pairs of electrons present in the oxime oxygen and amino nitrogen atoms to interact with the positively charged metal centre [114]. One study conducted by Zhao et al. explored the use of amidoxime-functionalized mesoporous silica-coated magnetic nanoparticles (MMS-AO) for U (VI) removal. The MMS-AO material, prepared through a co-condensation process, exhibited a maximum absorption capacity of 277.3 mg/g when tested in aqueous solutions [115]. Another approach, carried out by Bayramoglu et al., involved grafting polyacrylonitrile (PAN) onto MCM-41 silica using atom transfer radical polymerization (ATRP) and subsequently modifying it with amidoxime groups. The results showed that the adsorption capacity of the amidoxime-modified particles reached 442.3 mg/g, indicating a higher affinity of PAN-based amidoxime groups for U (VI). Furthermore, the silica-polymer hybrid adsorbent functionalized with amidoxime maintained 93% of the initial value even after ten regeneration tests [116]. Li et al. grafted and tested various groups onto mesoporous magnetic silica nanoparticles (MMSNs) to explore their potential for removing U (VI) from acidic or alkaline artificial groundwater. The groups tested included poly(propyleneimine) dendrimer (PPI) and polyamidoamine dendrimer (PAMAM). The objective was to evaluate their adsorption capacity and selectivity. The results demonstrated that the PPI-modified MMSNs exhibited the highest adsorption capacity, reaching approximately 133.3 mg/g at both pH = 3 and pH = 9.6. In contrast, when analysing the adsorption performance in a simulated water sample with a heterogeneous chemical composition containing Na 10,760, K 390, Mg 1280, Ca 410, Cl 19,380, SO_4_ 2910, and CO_3_ 140 mg/L, it was observed that PAMAM-modified MMSNs demonstrated the highest selectivity, with an adsorption capacity of approximately 53.8 mg/g [117,118]. In 2021, Abukhadra et al. synthesized an eco-friendly composite material (CH/MCM-48) by grafting chitosan polymer chains onto mesoporous MCM-48 silica. The resulting material exhibits a higher capacity for adsorbing U (VI) and Sr (II), common radioactive pollutants, from water. The integration process led to an increase in the surface area, reaching 587 m^2^/g, and introduced multifunctional active groups that enhanced the adsorption capacity. This silica/polymer hybrid material showed a promising retention capacity of approximately 260 mg/g for U (VI) and 330 mg/g for Sr (II), these values are higher than those of several organic and inorganic adsorbents. The adsorption process occurred through spontaneous and favourable exothermic reactions, with an equilibrium time of approximately 7 h. Moreover, the CH/MCM-48 composite material demonstrated significant recyclability for up to 5 cycles. It also exhibited a remarkable affinity for the studied radioactive ions, even in the presence of other metallic ions such as Cd (II), Pb (II), Zn (II), and Co (II) [119]. While Wang et al. enhanced the adsorption capabilities of nano-fibrous dendritic silica (DFNS) by grafting highly branched poly(amidoamine) (PAMAM) onto its surface. DFNS was chosen due to its large surface area, good stability, and highly porous structure which already exhibited effectiveness in removing U (VI). Compared to other silica or PAMAM-based materials, DFNS-PAMAM G2.0 showed faster adsorption kinetics, taking less than 180 min, and a higher adsorption capacity of 215.52 mg/g at 25 °C. The authors identified that the adsorption mechanism involved the complexation between amino/amidic groups present in the material and U (VI) ions. Thermodynamic parameters (ΔG°, ΔH°, ΔS°) indicated that the adsorption process is spontaneous and endothermic. Desorption and regeneration studies demonstrated that DFNS-PAMAM G2.0 exhibited regeneration capacity and maintained high efficiency for up to five cycles. These findings suggest that DFNS-PAMAM G2.0 holds great potential as an adsorbent for treating wastewater containing U (VI) [120].

In 2019, Sethy et al. used chitosan, a biocompatible, biodegradable, and non-toxic polymer, to create a hybrid nanocomposite capable of capturing chromium ions (Cr (VI)). In this work, chitosan was combined with poly (methyl methacrylate) (PMMA) through grafting, and cross-linking silica gel was added to enhance the mechanical strength of the nanocomposite. This process was carried out in a nitrogen atmosphere using ammonium persulfate as the initiator. Unlike traditional methods for removing Cr (VI), which are known to be inefficient and expensive, this new biocomposite proved to be both cost-effective and highly efficient. The adsorption of Cr (VI) was studied by varying the contact time between the adsorbate and the nanocomposite, the pH of the solution, and the doses of the composite. The optimal conditions for chromium removal were found at pH = 4, resulting in a remarkable 98% recovery from wastewater. Additionally, the biodegradability of the samples was studied at different time intervals, ranging from 15 days to 6 months, demonstrating their biodegradable nature [121].

A summary of the hybrid silica nanocomposites mentioned above is provided in Table 1 and Table 2.

### 3.2. Organic Dyes Remediation

To date, a vast number of organic chemical substances have been identified and produced, including pharmaceuticals, personal care products, pesticides, and organic dyes [27,122,123]. Unfortunately, many of these substances have characteristics that make them harmful to both human health and ecosystems. These substances are referred to as organic pollutants (OP) and can enter the environment through various sources, including industrial, agricultural, and domestic activities. Moreover, they have the capacity to persist in the environment for extended periods [124,125,126]. Typical examples of such pollutants include pharmaceuticals, pesticides, industrial dyes, halogenated compounds, aromatic compounds such as polycyclic aromatic hydrocarbons, and common industrial solvents [127,128]. Additionally, the escalating threat of CO_2_ emissions adds to these environmental concerns. The high presence of these organic pollutants in the environment poses a significant risk to the health of all living organisms and the environment itself. Hence, there is an urgent need to develop advanced technologies for their removal. Hybrid nanostructured mesoporous silica-based materials [129] functionalized with polymer-like molecular portions offer a promising opportunity for the removal of OP [46].

Among the major water pollutants, synthetic dyes find extensive usage in numerous industries, including plastics, paper, packaging, and notably, the textile and tanning sector [130,131,132]. Due to the significant risks they pose to human health and natural ecosystems, the exact annual production of dyes remains unknown. However, it is estimated that over 84k tons are discharged into the water each year [132], emphasizing the critical need for their removal. In the past, the removal of dyes has been achieved through the utilization of complexation reactions that can take place between suitably modified silanols surface and various dyes [133]. These mechanisms involve interactions such as hydrogen bonds, electrostatic and hydrophobic interactions, charge transfer interactions, or π-π interactions [133]. 

The incorporation of polymers as functionalizers of nanoparticles brings numerous benefits. The presence of different functional groups increases the capacity of nanomaterials for complex pollutants. Additionally, polymer functionality serves as a protective barrier, preventing direct contact between the silica surface and the acidic or basic dyes in water. Furthermore, the inclusion of the polymer component boosts the colloidal stability of the particles, thereby prolonging their contact time with contaminated water [46]. 

#### 3.2.1. Methylene Blue Adsorption

Methylene blue (MB) is an aromatic heterocyclic dye with numerous applications in the pharmaceutical and food industry. However, it possesses some negative characteristics, being carcinogenic and environmentally toxic due to its low biodegradability. In 2018, Zheng et al. [134] published a paper describing a rapid adsorption technique for methylene blue using a material created through the grafting of acrylic acid (AA) and 2-acrylamido-2-methyl-1-propanesulfonic acid (AMPS) onto the surface of vinyl-modified silica magnetic nanoparticles. The results showed an adsorption capacity of 421.9 mg/g, which directly correlated with the pH of the solution and the amount of polymer attached to the nanoparticles. The adsorption process was attributed to both electrostatic and hydrophobic interactions, as well as hydrogen bonds. Electrostatic interaction occurs between the deprotonated groups (-COO^−^ and -SO_3_^−^) on the nanomaterial’s surface and the positively charged quaternary ammonium groups in MB. Additionally, hydrogen bonding formation takes place through the interaction between -OH and -NH groups on the surface of the material and the amine groups present in MB [135,136]. Hydrophobic interaction refers to the tendency of nonpolar groups to associate with each other in an aqueous solution. In the case of the grafted AMPS, its hydrophobic character arises from the presence of aliphatic branches, enabling it to interact with the benzene rings in methylene blue through hydrophobic interactions [137,138]. At the end of the adsorption process, the authors regenerated the system by subjecting it to an acid treatment. This treatment preserved 60% of the adsorption capacity, enabling the nanomaterial to be reused for up to eight consecutive cycles. In a specific study focusing on methylene blue adsorption, Zakaria et al. synthesized polyglyceroldimethacrylate (poly(GDMA)) using glycidyl methacrylate (GMA) and methacrylic anhydride in a solvent-free process and with the aid of a green catalyst (Bentonite-H). The resulting poly(GDMA) was then used to functionalize MCM-41 through an in situ free radical polymerization reaction initiated by benzoyl peroxide (BP). Different hybrid materials with different percentages of bound poly(GDMA), named NC1, NC2, and NC3, were synthesized, with percentages of 19.12%, 36.41%, and 47.48%, respectively. Despite NC3 exhibiting poorer structural and texture qualities, due to the dispersion of the polymer within the pores, it showed the best adsorption results (111.11 mg/g). This suggests that a higher number of binding sites correlates with increased interactions with the cationic dye [139]. In 2019, Torad et al. worked on KIT-6 nanoparticles, functionalizing them with β-cyclodextrin using a post-grafting technique. The results showed an enhanced methylene blue adsorption capacity of the nanosystem compared to unmodified KIT-6, achieving adsorptions of 149.6 mg/g, approximately 1.6 times higher. This enhanced adsorption can be attributed to the expanded and uniform pore channels, creating favourable conditions for the complete penetration of MB molecules towards the active binding sites of β-CD. This promotes effective host-guest interactions between β-CD and MB molecules within the hydrophobic inner cavity of β-CD, leading to the formation of stable host-guest inclusion complexes [140,141]. The adsorption capacity and efficiency exhibited a strong correlation with the pH of the medium employed [142]. In 2020 Saleh et al. modified commercial silica nanoparticles using acrylic acrylamide (SAA). They demonstrated the capability of this hybrid nanosystem to adsorb a large amount of MB, achieving an adsorption capacity of 375.9 mg/g under optimal reaction conditions. These conditions included an initial dye concentration of 10 mg/L, a contact time of 30 min, pH = 6, and an adsorbent dosage of 50 mg/30 mL. The research highlighted that the key factors influencing the adsorption capacity were the initial pH, followed by the adsorbent dosage, contact time, and an increase in initial concentrations. Furthermore, the thermodynamic study indicated that the adsorption reaction becomes more spontaneous with higher temperatures. The adsorption process can be attributed to an intricate complexation mechanism involving specific functional groups such as carbonyl, amide, hydroxyl, and silica groups present in the surface-active agent (SAA) and the nitrogen (N) and sulfur (S) atoms found in the methylene blue molecules. Moreover, the presence of π-π conjugation and π-π stacking interactions can further enhance the adsorption [143,144]. In 2019, Abid et al. focused their research on SBA-15 mesoporous silica functionalized with polyvinyl alcohol (PVA) to adsorb methylene blue. They successfully obtained nanocomposites with varying percentages of loaded polymer. Their findings revealed that the adsorption capacity decreased beyond a certain concentration of PVA, likely due to the occlusion of SBA15 nanostructure pores. The sample with a 30% loading showed the highest adsorption capacity. The authors also investigated the effect of additional parameters, such as contact time, adsorbent mass, and initial dye concentration, on the adsorption process, concluding that the main mechanism of methylene blue adsorption on the PVA/SBA-15 composite is through electrostatic interactions. This occurrence is likely a result of modifying the surface charge state and enhancing interactions between the polymer’s functional groups and the MB dye. The authors suggest that it would be useful to investigate the effectiveness of these materials in adsorbing other types of pollutants, including heavy metals, phenols and their derivatives, anionic dyes, as well as gases such as volatile organic compounds (VOCs) and carbon dioxide (CO_2_) [145]. Several studies, including Jadhav et al. [146], have shown the usefulness of surface functionalization of silica nanoparticles with polymeric components. This functionalization not only facilitates effective adsorption of pollutants but also enables easy desorption, making these nanoparticles suitable for application in sample preparation analytical techniques, as well as the recovery and concentration of analytes from complex matrices. Jadhav et al. used a thermoresponsive polymer, poly(N-isopropylacrylamide), which can switch the surface characteristics of nanoparticles from hydrophilic to hydrophobic upon heating. This property allows for the release of captured pollutants such as methylene blue, caffeine and amoxicillin, enabling easy regeneration of the absorption substrate by adjusting the temperature. Furthermore, hybrid/composite mesoporous silicas have been employed for the adsorption of several different toxic dyes, including Methyl Orange, Bromothymol Blue, Congo Red, Sunset Yellow, and Acid Blue 62. Boukoussa and his colleagues [147] focused on the adsorption of Methyl Blue (MB) and Methyl Orange (MO), the latter being an aromatic azoic dye with mutagenic properties. They modified the surface of SBA-15 through in situ functionalization with polypyrrole, achieved by surface adsorption of distilled and liquid polypyrrole. They also evaluated the adsorption performance of the functionalized nanocomposites with different polymer amounts, and measured contact times. The results revealed that both dyes exhibited significant adsorption within the initial few minutes, indicating a strong affinity between the nanocomposite and the dyes. The adsorption process of both dyes was primarily governed by chemisorption, which can be attributed to the various interactions between the dyes and the functional groups of the adsorbents. These findings are consistent with previous studies [148,149,150]. In addition, the surface modification significantly affects the affinity of the adsorbent towards MO, primarily due to the presence of amine groups. Acid/base reactions occur between the acidic MO and the basic PPy/SBA-15. Furthermore, even at lower pH levels, there is observed adsorption, that can be due to alternative interactions, such as van der Waals forces, or hydrogen bonding between MB and the functional groups on the adsorbent’s surface. The best results were obtained with 50% functionalization for Methyl Orange and 1% functionalization for Methylene Blue, probably due to the acidic nature of silanol groups that can readily interact with the latter dye. In terms of pH, it was found that MO displayed the highest adsorption at pH = 6, while MB exhibited the highest adsorption above pH = 4, confirming an electrostatic interaction mechanism [147].

The adsorption of Methylene Blue (MB) using polymer-modified nanoparticles is an active and promising research area. The functionalization of silica nanoparticles with these polymers enhances their adsorptive properties, enabling them to efficiently capture methylene blue from aqueous solutions.

The presence of these polymers allows for various types of interactions with MB, including electrostatic, hydrophobic, hydrogen bonding, and complexation interactions. These interactions are crucial for the effective adsorption of the dye and depend on factors such as the solution pH, the amount of polymer bound to the nanoparticles, and the chemical structure of the polymers themselves. 

The results of these studies indicate that polymer-functionalized silica nanoparticles exhibit significantly improved adsorption capacity compared to unmodified nanoparticles. The amount of dye adsorbed depends on experimental parameters such as pH, initial dye concentration, contact time, and adsorbent dosage. Furthermore, some studies highlight that the functionalization of nanoparticles with polymers enables the easy desorption of captured contaminants, contributing to the regeneration of the adsorption substrate.

#### 3.2.2. Adsorption of Methyl Orange and Other Dyes

In 2021, Alotaibi et al. conducted a study focusing on the adsorption of Methyl Orange (MO) and Sunset Yellow (E110) dyes. To achieve this, they used mesoporous silica magnetic nanoparticles, modified with poly(2-diethylaminoethyl methacrylate) (PDEAEMA) using the atom transfer radical polymerization (ATRP) technique. In order to control the external charge of PDEAEMA, the tertiary amine groups on the polymer chains were quaternized through treatment with 2-iodoethanol, resulting in a permanent positive charge on the modified nanoparticles (Fe_3_O_4_@MSN-QPDMAEMA). This modification allowed for interactions with the negatively charged anionic dyes. The study also investigated the influence of pH, initial dye concentration, and contact time on the adsorption capacity of the hybrid nanosystem. The authors demonstrated that quaternization had a positive effect on the adsorption capacity compared to the non-quaternized nanocomposite, achieving adsorption capacities of 294 mg/g and 194.8 mg/g for MO and E110, respectively. It is important to note that these adsorption results are particularly significant considering the ease of synthesis, low cost, and abundance of the starting materials used to create the hybrid nanocomposite [151]. Similarly, Beagan et al. have developed a system based on magnetic silica nanoparticles functionalized with [poly(2-methacryloyloxy)ethyl] trimethylammonium chloride solution (PMETAC), which is effective for the adsorption of Methyl Orange and Bromothymol Blue dyes. The functionalization process was achieved through the atom transfer radical polymerization (ATRP) technique, resulting in spherical nanoparticles with a magnetic core and an average size of approximately 30 nm. The researchers found that the adsorption capacity of the functionalized nanoparticles was unaffected by the pH range studied (pH = 3 to pH = 9), and the adsorption efficiency for both dyes was 100% [152]. These studies demonstrate that the functionalization of nanoparticles with positively charged polymers enhances the adsorption efficiency of anionic dyes by facilitating interactions with their negative charges. Positive results were obtained in terms of adsorption capacity, and the polymer functionalization techniques used offer advantages in terms of ease of synthesis and availability of starting materials.

On the other hand, in 2017, SBA-15 functionalized with poly(amidoamine) (SBA-15/PAMAM) in dendritic form was developed for the adsorption of Acid Blue 62 (AB62), an anionic dye. The adsorption behaviour of the functionalized material was carefully investigated, revealing that the ionic interactions between the amine functionalities of the dendrimers and the anionic sulfonated groups on the dyes played a crucial role in the adsorption process. By evaluating various factors including pH, adsorbent concentration, contact time, and dye concentration, the authors identified the best adsorption conditions: pH = 2, an adsorbent concentration of 0.03 g/L, and a contact time of 60 min at 25 °C. The maximum adsorption capacity achieved was 1428.57 mg/g [153]. These findings highlight the potential of utilizing functionalized SBA-15/PAMAM for the effective adsorption of anionic dyes.

Torabinejad et al. studied the modification of MCM-41 with polypyrrole (PPy) and polyaniline (PAni) and compared the efficiency of these two materials. The functionalization of MCM-41 with both polymers was achieved through chemical polymerization techniques. The results of the study showed that in terms of adsorbing AB62, the nanocomposite polymerized with Polypyrrole exhibited higher efficiency compared to the one functionalized with polyaniline, probably due to the smaller molecular size of polypyrrole. The adsorption of the dye was found to be inversely dependent on the pH of the solution. Furthermore, the adsorption capacity increased with higher temperature, longer contact time, and higher amounts of adsorbent dosage. The optimal conditions for adsorption were determined as pH = 2, an adsorbent quantity of 0.1 g, a contact time of 90 min, and a dye concentration of 20 mg/L. Under these conditions, the efficiency obtained was 90.57% for PPy/MCM-41 and 38% for PAni/MCM-41. The observed adsorption mechanisms involve the formation of ionic bonds between the cationic functional groups present in the adsorbents and the anionic functional groups present in the dyes [154].

N.N. Harsini et al. reported an interesting advancement in the utilization of polymer-grafted silica nanoparticles. They demonstrated the adsorption of the dye Congo Red, a known carcinogen and pollutant, using a molecularly imprinted polymer (MIP) on silica magnetic nanoparticles. The surface modification of the nanoparticles is achieved with aminopropyltriethoxysilane (APTES) to enable selective interaction with the target dye. The presence of a MIP is able to selectively interact with the dye to be adsorbed, removing even a single dye from a mixture. The MIP coating is achieved through coprecipitation with acrylamide as the functional monomer. The results showed high adsorption efficiency, reaching a maximum value of 22.76 mg/g, acceptable selectivity, and the possibility of reusing the functionalized nanosystem for at least three cycles [155]. 

In 2018, Ghanei et al. worked on the removal of Acid Blue 25 dye. They studied the application of magnetic SBA-15 functionalized with cross-linked poly(acrylic acid) (CPAA) polymer. The authors investigated the influence of various parameters, including pH, temperature, contact time, dye concentration and adsorbent concentration on the adsorption process. The results revealed good adsorption efficiency, with a maximum of 909.09 mg/g obtained using an adsorbent dosage of 0.025 g, a contact time of 60 min at 25 °C and a pH = 2. The positive charge on the adsorbent surface at pH = 2 facilitates the interaction with the negatively charged dye molecules, thanks to the presence of sulfonic groups (−SO_3_–) on the adsorbent. [156]. In a 2017 study by Tugce A. Arica et al., magnetic MCM-41 silica particles were modified to possess magnetic properties. The modification process involved several steps to graft poly(glycidyl methacrylate) (pGMA) onto the surface of the particles. These polymer chains served as anchors for tris(2-aminoethyl) amine (TAEA) molecules, and the polymerization was carried out using the atom transfer radical polymerization (ATRP) technique. The researchers investigated the application of these nanocomposites for the adsorption of Direct Blue 6 (DB-6) and Direct Black 38 (DB-38), which are both considered carcinogenic and prohibited for use in the textile industry by the EU (2002/371/EC decision) [157]. The results demonstrated a good adsorption efficiency, with removal capacities of 142.7 mg/g for DB-6 and 79.9 mg/g for DB-38. The observed interaction mechanism involved the formation of hydrogen and electrostatic bonds between the amino groups on the particle surface and the sulfonic groups present in the dyes [158].

Abdullah M. Alswieleh used zwitterionic polymers for the adsorption of Rhodamine B (Rh B) and Crystal Violet (CV) dyes. These polymers, derived from poly(2-(tert-butylamino) ethyl methacrylate), were employed to create a composite system known as PTBAEMA−SO3H@MSNP. The research findings revealed that the efficiency of the removal process increased with longer exposure time but decreased as the concentrations of both dyes increased. The optimal pH for the removal of Rh B was found to be pH = 7, while pH = 9 was optimal for CV, owing to the electrostatic interaction between the polymer chains and the dye molecules. At these conditions, the removal efficiency was approximately 68% for Rh B (at 200 ppm) and 90% for CV (at 400 ppm). The adsorption of both dyes on the PTBAEMA-SO_3_H@MSNP adsorbent occurred spontaneously, exothermically, and with an increase in entropy [85].

Moreover, MSN (Mesoporous Silica Nanoparticles) can improve the chemical-physical properties of polymeric nanocomposites, making them suitable for environmental applications. Guerritore et al. developed hybrid nanocomposites called Hyper-Crosslinked Polymer, by incorporating mesoporous silica nanoparticles (MSN) into vinylbenzyl chloride and divinylbenzene to capture polar dyes from water. As a result, the nanocomposites showed a higher surface area and greater porosity compared to the Hyper-crosslinked resins without MSN. The system was applied to the adsorption of Remazol Brilliant Blue R, and the results indicated that the nanocomposite containing five phr (parts per hundred rubber) of MSN displayed the best adsorption performance, as well as the highest surface area (1824 square meters per gram) [159]. Bahalkeh et al. conducted research on the removal of Brilliant Red E-B4A from water. This cationic dye, belonging to the triphenylmethane family, is soluble in both water and ethyl alcohol and possesses an aromatic structure that hinders biodegradation. Therefore, the development of an adsorption method for its removal from contaminated water sources is highly recommended. To address this, the researchers functionalized SBA-15 with chitosan, a natural and eco-friendly polymer known for its high adsorption capacity due to the presence of numerous amino groups in its molecular structure. They compared the adsorption capacity of the functionalized SBA15 with that of the unfunctionalized version and evaluated the effects of pH, contact time, and adsorbent concentration. The observed results demonstrated that the nanocomposite obtained is an effective adsorbent for this dye, with the optimal adsorption conditions being pH = 7, 0.03 g of adsorbent, and 40 min of contact time. The suggested adsorption mechanism involved strong electrostatic attractions between protonated chitosan and a sulfonic acid group of the brilliant red E-4BA dye. Additionally, hydrogen bonds between hydroxyl and amine groups of chitosan, SBA-15, and brilliant red E-4BA can also contribute to the adsorption process. The mesoporous structure of SBA-15 also contributed by physically capturing dye molecules and increasing the dye removal capacity of the synthesized adsorbent [160]. Aghajani et al. focused on the adsorption of Reactive Orange 16 (RO 16), an azo-dye that is difficult to remove due to its solubility in water. They used SBA-15 modified with polyaniline, a material known for its ease of preparation and low synthesis cost. The polymerization of aniline on SBA-15 was achieved chemically in an acidic solution using an oxidizing agent such as KIO_3_. The results obtained under optimal conditions (pH = 2, contact time of 60 min, adsorbent concentration of 0.4 g/L) were encouraging, demonstrating the effectiveness of the nanocomposite in adsorbing the dye [161].

In Table 3 is summarized the Hybrid systems described.

### 3.3. Remediation of Other Organic Compounds 

#### 3.3.1. Drugs 

Pharmaceutical compounds such as analgesics, anti-inflammatory drugs, and antibiotics, play a crucial role in people’s lives [162]. Over the past few decades, their consumption has increased worldwide [163]. Unfortunately, these compounds can enter environmental matrices through various pathways, including human excretion, improper disposal, leaching from soil, drainage, or industrial activities. Pharmaceutical residues have been detected in urban and hospital wastewater, surface water, and groundwater, posing a potential threat to aquatic species and human health due to their toxicity [164].

Achieving complete elimination of pharmaceutical compounds using conventional treatment methods is challenging since they are typically present at low concentrations in the range of µg/L or ng/L [165]. Therefore, there is a pressing need for the development of new techniques and materials to remove these compounds effectively. The utilization of appropriately functionalized nanostructured materials, such as Molecularly Imprinted Polymers (MIPs), previously discussed in the section on dye removal, can provide practical applications for drug removal [166,167].

Mehdinia et al. used SBA-15 as mesoporous silica material for the synthesis of molecularly imprinted polypyrrole. The resulting nanostructured material was then utilized for the adsorption of ascorbic acid from aqueous solutions. In order to demonstrate its selectivity, non-competitive and competitive tests were carried out, using similar molecules such as dopamine, paracetamol, and epinephrine. Traditional use of MIPs often presents some drawbacks, such as incomplete removal of the template, low binding capacity, or irregular material shape. However, by using SBA-15 as a substrate, the authors successfully addressed these issues, resulting in MIP-SBA15 hybrid materials that acted as effective adsorbents for ascorbic acid. The obtained material showed excellent recognition and adsorption capacities for ascorbic acid, even in the presence of competitive targets, thereby highlighting the remarkable selectivity of this nanocomposite [168].

The application of MIP for the removal of environmental pollutants is extremely interesting given the level of specificity that can be achieved. In 2018, Fu et al. investigated the use of MIP for the removal of 17β-Estradiol from the environment. 17β-Estradiol is one of the several naturally occurring estrogens, and its persistence and tendency to accumulate pose significant risks to the environment and various animal species. Although numerous techniques have been employed to eliminate it from the environment, challenges such as slow adsorption and a lack of selectivity remain unresolved. The specific MIPs developed for 17β-Estradiol showed remarkable adsorption specificity and a detection capacity of 0.01 micromoles/litre [169]. Furthermore, MIP can be also immobilized on mesoporous silica supports.

Recently, Xu et al. carried out a study focused on the removal of salicylic acid from the environment using nanocomposites functionalized with MIP. Salicylic acid finds application not only in the pharmaceutical field but also in the cosmetics and preservatives industry, representing a toxic threat to both the environment and human health. Therefore, the development of suitable technologies for its capture is of great interest. The developed MIP were immobilized on SBA-15 supports. Chemical-physical characterization revealed the highly ordered mesoporous structure of the supporting nanoparticles, displaying a high adsorption capacity and excellent selectivity towards salicylic acid. The data demonstrated the influence of pH, initial salicylic acid concentration, and temperature on the adsorption capacity. The optimal conditions were observed at pH = 4.62, a salicylic acid concentration of 52.38 mg/L, and a temperature of 35 °C. The studies also showed the possibility of carrying out multiple adsorption-desorption cycles (at least 5) without significant loss of adsorption capacity [167].

Another example showing the application of polymer-grafted nanomaterials for the removal of Pharmaceutical Pollutants (PP) from water can be found in the work of Alfhaid. The study investigated a method to remove paracetamol (PCM) from aqueous solutions using mesoporous silica nanoparticles (MSN) functionalized with pH-sensitive polymer portions of poly(2-(start-butylamine) ethyl methacrylate) (PTBAEMA). The functionalization process was carried out using the ATRP technique, both on the internal and external surfaces of the nanoparticles. The authors demonstrated how PCM adsorption was influenced by the pH value, obtaining an optimal uptake result of approximately 237 mg/g. The maximum adsorption efficiency was calculated at 92% when the pH values ranged from 5 to 7. An increase in pH significantly reduced the adsorption capacity. The reported results were also affected by the contact time and likely involved π-π interactions [170].

**Figure 3:**
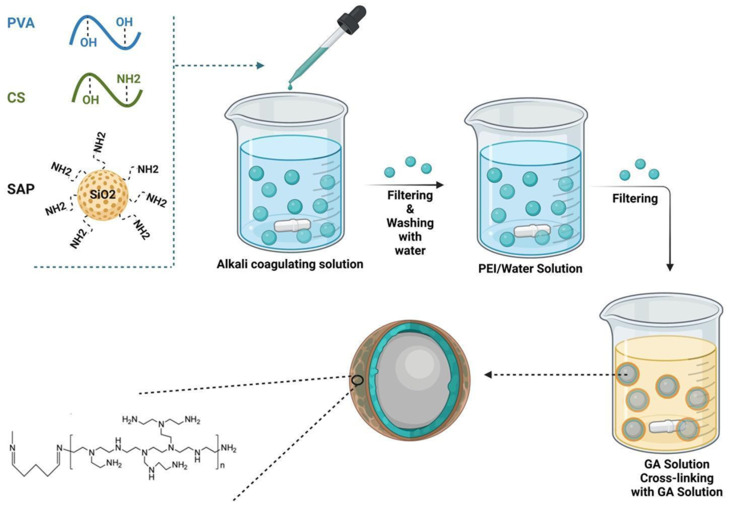
Preparation of core-shell/bead-like PVA/CS/SAP@PEI composites [171].

Yuqing Lu et al. developed a nanostructured material based on polyvinyl alcohol and chitosan-modified silica nanoparticles for the recovery of diclofenac from water. A schematic representation of the development process is presented in Figure 3. Diclofenac is one of the most extensively used drugs worldwide and poses challenges for water treatment due to its multiple transformation by-products [171]. The authors observed the occurrence of an interaction between the amine groups of the nanomaterial and the carboxylic groups on diclofenac, particularly under acidic conditions. The maximum adsorption was found at pH = 5, reaching 493.81 mg/g [171].

Table 4 summarizes the hybrid systems described.

#### 3.3.2. Oil-Derived Organic Compounds and Polycyclic Aromatic Hydrocarbons (PAHs)

As a result of the significant growth in crude oil and gas production, a substantial amount of produced water (PW) containing heavy metals [172] and organic substances, including hydrocarbons, is generated, posing a severe threat to both the environment and public health. Thus, it is crucial to develop advanced methods to treat, recover, and reuse such contaminated wastewater to prevent the release of non-degradable substances into the environment.

In a study conducted in 2021, Elmobarak and Almomani investigated the use of mesoporous silica-based nanomaterials for adsorbing oil from produced water and creating a de-emulsifying material. They achieved this by functionalizing the nanostructured supports with hyperbranched polyglycerol chains. Initially, Fe_3_O_4_-SiO_2_ magnetic nanoparticles (Fe-Si-MNPs) were synthesized, and then polyglycerol chains were added to obtain the de-emulsifying material, called PSiMNPs. The results obtained showed that by adding only a small percentage of material to an O/W (oil in water) emulsion, the demulsification capacity reached an efficiency of 90%, and this was accomplished within a relatively short time (about five minutes for a petroleum concentration of 500). Furthermore, the magnetic properties of the material enabled easy recovery of the support through the application of a simple magnetic field, allowing for the reuse of the same material up to 15 times, as indicated by the obtained data. The authors also optimized various parameters, such as oil concentration, de-emulsifying dose, pH, and salinity to evaluate the adsorption process [173].

The impact of oil spills on aquatic life and ecosystem recovery highlights the need for effective methods to disperse oil agglomerates. Amphiphilic-grafted nanoparticles (AGNs) have emerged as a potential solution, given their promising ability to encapsulate oil. In a recent study, Keller et al. investigated the oil entrapment performance of AGNs consisting of a silica nanoparticle core and grafted PCL-b-POEGMA copolymer. Their findings indicated that these AGNs exhibit higher effectiveness at lower concentrations compared to the widely used surfactant Corexit. The AGNs demonstrated the capability to encapsulate PAHs at concentrations as low as 0.0015 mg/mL without significant loss in efficiency. However, the ratio of aromatic to aliphatic hydrocarbons present in the oil plays a significant role in the encapsulation process, with AGNs exhibiting a preference for PAHs due to their greater availability in the aqueous phase. The hydrophilic to hydrophobic polymer content ratio has a greater impact on oil uptake than the length of the polymer chains. Nevertheless, achieving the desired properties required the use of minimum molecular weights for both PCL and POEGMA. These outcomes suggest that AGNs are environmentally friendly and offer a promising alternative to Corexit, particularly when used at lower concentrations [174].

Recently, the application of polymer-coated nanoparticles has emerged as a promising approach to improve the efficiency of the enhanced oil recovery (EOR) technique. This is primarily attributed to their solubility, emulsion-stabilizing ability, and their capacity to reduce particle retention on the rock surface. Alberto Bila et al. conducted experiments using five different polymeric-coated nanomaterials to enhance the stability of these nanomaterials in seawater, which otherwise would not have withstood the high concentrations of Ca^2+^ and Mg^2+^ salts. These functionalized nanomaterials were used as additives in seawater injection for oil recovery in neutral-wet Berea sandstone. The results demonstrated an increase in oil recovery ranging from 2.5% to 16%, thereby highlighting the effectiveness of the approach [175].

The use of silica nanomaterials modified with polyacrylamide (PAM) has also been proposed for enhanced oil recovery (EOR). However, when exposed to high temperatures and salinity, these materials tend to agglomerate. To address this issue, Haruna et al. explored the modification of the material by incorporating APTES (3-aminopropyltriethoxysilane). This modification enables the material to acquire a positive surface charge, which facilitated the stabilization of the dispersion through strong coordination bonds between MSN-APTES and PAM, which possesses a negative surface charge. The authors successfully demonstrated the stability of MSN-APTES/PAM under rigorous operating conditions and highlighted its superior performance in enhancing oil recovery compared to the use of free polymer alone [176].

On the other hand, Vo et al. reported the application of Poly(methacrylic acid)-functionalized SBA-15, synthesized from co-condensation of tetraethoxysilane with varying amounts of 3-(trimethoxysilyl)propyl methacrylate in an acidic medium, for the adsorption of phenol in water. Phenol, an extremely toxic aromatic hydrocarbon derived from industries such as oil refining, petrochemical production, ceramic manufacturing, coal conversion, and the phenolic resin industry, requires efficient removal from the environment. Among different techniques attempted for its elimination, adsorption offers the most advantages and the lowest costs. The results obtained from the use of this type of functionalized nanomaterial revealed that the adsorption of phenol is significantly influenced by pH, with increased adsorption occurring at lower pH levels. The adsorption process mainly involves the interaction between the proton of phenol and the carbonyl and carboxyl groups present on the nanomaterial [177]. A significant number of hydrocarbons represent a high risk to the environment and human health, including polycyclic aromatic hydrocarbons (PAHs). PAHs comprise a large group of polluting organic compounds, characterized by the presence of at least one benzene ring (making them aromatic) and exhibiting carcinogenic, teratogenic, and mutagenic properties [178].

The presence of PAHs in the environment is widespread and represents a serious problem, especially in close proximity to urban areas where their levels tend to be higher. Human exposure to PAHs can occur through various sources such as cigarette smoke, food, and drinking water. Due to their lipophilic nature and widespread presence in water sources, PAHs can easily accumulate in tissues and exert their long-term effects in vivo [179]. 

Arkas and Tsiourvas carried out research on the adsorption of PAHs in water, using silica nanocomposites modified with hyperbranched polyethyleneimine dendrimers. The study revealed that this type of nanocomposite is capable of effectively adsorbing PAHs from water, through a combination of electrostatic interactions and the chelating capacity of the polymer. Additionally, charge transfer interactions facilitate interaction between PAHs and the nanostructure. The adsorption results achieved by the synthesized nanocomposites can be attributed to the formation of charge transfer complexes between the tertiary amino groups and the polycyclic aromatic compounds [180].

Although they are hydrophobic, PAHs are soluble in water in small quantities. They have been found in groundwater (1.0–10.0 ng/L), rainwater (2.7–7.3 ng/L), tap water (2.5–9.0 ng/L), and surface waters (10–830 ng/L). Topuz and Uyar developed a new nanostructured material based on silica nanoparticles functionalized with cyclodextrin. Given its structure, cyclodextrin is capable of using its outer part to create bonds with lipophilic molecules and its inner part to bind hydrophilic molecules, thanks to primary and secondary hydroxyl groups. The functionalization occurred by condensation in a one-pot reaction during the synthesis of mesostructured silica nanoparticles. The results show that the adsorption capacity of PAHs is comparable to that of activated carbon filters, which are commonly used to remove these pollutants from water. Further studies are underway to evaluate the reusability of the supports produced [181].

#### 3.3.3. Removal of Gaseous Compounds, CO_2_ and Other Gases

Around 70% of the greenhouse gases emitted into the atmosphere are produced by human activities. CO_2_ is a potent greenhouse gas and the main cause of climate warming according to the IPCC [182,183,184,185]. In order to keep the temperature, increase below 2 °C, and preferably within 1.5 °C it is not enough to simply reduce further carbon dioxide emissions into the atmosphere, but it is necessary to find ways to capture and store it in a way that makes it unavailable for re-emission into the atmosphere [186].

There are several methods for capturing carbon dioxide and they can be classified into 4 major techniques: pre-combustion, post-combustion, oxy-fuel combustion, and electrochemical separation. [187,188,189]. Since the 1930s, liquid amines have been used in scrubbers for the separation of CO_2_ from other gases such as natural gas and hydrogen, although the first actual applications were in the 1990s [190]. Many of the amines used are Alkanolamines, including monoethanolamine (MEA), diethanolamine (DEA), methyldiethanolamine (MDEA) and piperazine (PZ). These molecules have been extensively studied and, the projections show that liquid amine scrubbers will remain the dominant technology for CO_2_ capture until at least the 2030s. However, this technology is highly energy-consuming, especially given the high temperatures required in the recovery processes. Moreover, these processes also have other disadvantages: amine solutions are subject to oxidative degradation, carbamate polymerization, and thermal evaporation. The degradation rate of amines in the presence of sulfur dioxide (SO_2_) further pushes for additional degradation, resulting in the formation of toxic and polluting gases such as nitrosamines, nitramines, and nitric acid [190]. Immobilizing these compounds that have a high affinity for carbon dioxide to mitigate the negative aspects of their application is an interesting field of research. Due to their characteristics of high CO_2_ adsorption capacity, low energy consumption, reduced corrosion, and stability in gaseous contaminants, amine-based adsorbents are highly attractive for CO_2_ capture processes. For example, numerous studies in the literature [191,192] describe the functionalization of silica nanostructures modified with amine functional groups, which are very useful for CO_2_ capture.

Yu et al. have worked on silica nanostructures modified with melamine-based dendrimers. They obtained nanocomposites with variable sizes ranging from 2 to 30 nm, and surface areas ranging from 68.2 to 614.9 m^2^/g. Comparative results between the dendrimer-modified nanoparticles and the unmodified ones were studied, and relevant adsorptions were observed for the dendrimer-modified nanomaterials compared to the unmodified ones. According to the authors, this is due to both the larger surface area of the nanomaterial and the presence of a higher number of CO_2_-philic functional groups in the dendrimer-modified material compared to the amino-modified one [193].

Sanz et al. demonstrated how the use of nanoparticles belonging to the SBA-15 family, impregnated with PEI, allowed for significant capture of this gas. They demonstrated that the CO_2_ adsorption mechanism is based on chemisorption and that the concentration of PEI impregnated on the nanomaterial’s surface was an important variable in obtaining good results, along with temperature, which was evaluated in the range between 25 and 75 °C. The maximum CO_2_ sequestration obtained was 90 mg/g, at 75 °C and one bar. This result is consistent with the claim that the adsorption mechanism is of a chemical type. The polymer-coated material was studied by applying different adsorption-desorption cycles, always achieving excellent adsorption efficiency. According to the researchers, the material can be used for multiple cycles [194]. The work of Li et al. was also based on the use of PEI. Their results showed, consistent with the previous work, that CO_2_ adsorption was correlated with both temperature and the amount of PEI impregnated on the nanomaterial. They demonstrated how the capture of CO_2_ increased with an increase in the amount of PEI. However, a decrease in CO_2_ diffusion was observed when the amount of impregnated PEI increased too much, due to pore closure on the nanomaterial. According to the researchers, increasing the temperature could overcome this problem, allowing for better diffusion of the CO_2_ to be captured. The best results obtained by the research group were seen under these conditions: 60% of PEI impregnated on the nanomaterial and an adsorption temperature of 105 °C. Regarding the adsorption/desorption cycles necessary for the reuse of the nanostructured system, the researchers showed how the use temperature is a fundamental parameter to be kept under control, as above 105 °C, there is a considerable risk of urea production and, therefore, PEI leaching during adsorption/desorption cycles [195].

Mesoporous silicas containing supported amine groups exhibit a strong affinity for carbon dioxide due to the adsorption sites provided by the -NH_2_ groups (CO_2_-NH_2_-R interaction). However, the access to the internal surface of these silicas is limited by CO_2_ condensation at the pore entry, and the basicity of the surface can lead to the formation of carbamates, making CO_2_ desorption challenging without heat treatment that may result in amine loss through evaporation and degradation. To address this issue, organic moieties that impart hydrophilic properties can be grafted onto the material to enhance its adsorptive capabilities. Ghomari et al. achieved this by functionalizing MCM-48 with polyol H_2_0 dendrimers, resulting in a significant improvement in CO_2_ affinity with a CO_2_ uptake of up to 320.5 umol/g. The dendrimer was found to be responsible for the enhanced CO_2_ retention, as the MCM-48 framework showed weak interaction towards CO_2_. Notably, the dendrimer also improved the adsorption and release properties of CO_2_. The release of CO_2_ was achieved by heating at temperatures below the thermal stability limit, and repetitive adsorption-desorption cycles did not affect the affinity of the material towards CO_2_ [196].

In a study conducted by Boukoussa et al., mesoporous SBA-15 silica was prepared and modified through in-situ polymerization of varying amounts of aniline, resulting in nanocomposites referred to as Polyaniline/SBA-15 (PAni/SBA15). These nanocomposites exhibited a high affinity towards CO_2_, and the study focused on achieving the lowest possible adsorption and desorption temperatures for sustainable CO_2_ capture technology. The surface basicity and hydrophilic character of the PAni/SBA-15nanocomposites were examined using temperature-programmed desorption of CO_2_ and H_2_O. The in-situ polymerization of aniline allowed the successful preparation of the PAni/SBA-15 nanocomposite without causing any structural swelling, but a decrease in pore size was observed due to the formation of a thin film of polyaniline [84].

**Figure 4:**
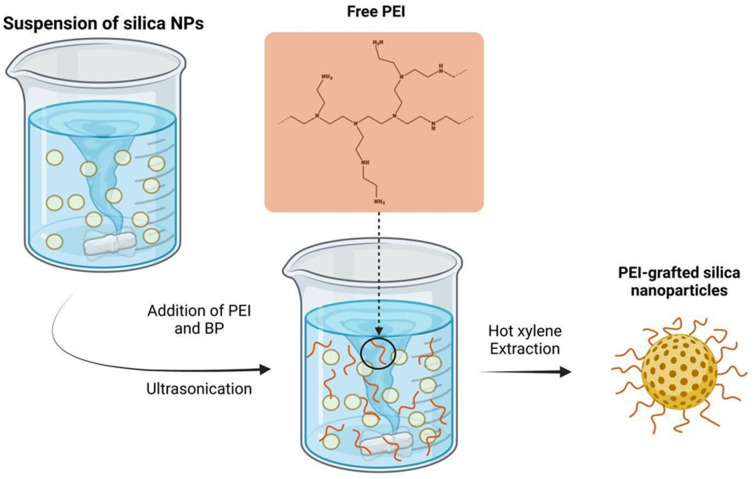
Grafting of Poly(ethylene imine) to silica nanoparticles for odour removal from recycled materials [197].

PEI grafting is not only used for CO_2_ capture. Cohen et al. [197] have also applied the use of modified nanomaterials for odour removal from recycled plastics. The main limitation of the reuse of recycled plastics is the unpleasant odour emitted by polymers and composite materials. Some studies have shown that recycled HDPE from household waste contains at least 70 different types of substances, including limonene [198]. Typically, plastic materials are added with an odour-neutralising compound during recycling. However, the authors have shown that the use of silica nanoparticles modified with PEI, as can be seen in Figure 4, even at very low concentrations, allows for a significant reduction in the unpleasant odour in recycled plastics, enabling greater reuse of plastic and composite materials [197].

Machowski et al. used silica nanoparticles belonging to the MCM–M41s family, with a spherical shape and a different pore structure arrangement. They then functionalized the surfaces with polyfurfurylalcohol through a precipitation reaction and subsequent activation at 520 K. They applied this hybrid nanomaterial to the adsorption of vapours of various volatile ketones, such as acetone, methyl-ethyl ketone, and methyl-isobutyl ketone, and compared it to the adsorption using unmodified nanoparticles. They demonstrated that the functionalized material allows for better adsorption of these ketones, possibly due to the presence of carbonyl groups that interact with the ketones via hydrogen bonding, forming enolic and non-ketonic forms, unlike the hydroxyl groups present on the unmodified silica nanoparticles [199].

#### 3.3.4. Other Organic Compounds

The use of functionalized silica-based nanostructured materials can also be applied to other classes of organic pollutants. Alotaibi showed in 2021 how the use of pH-responsive polymer-functionalized silica nanoparticles have the ability to adsorb the herbicide 2,4,5-trichlorophenoxyacetic acid (TCA) from polluted water. The nanomaterials were functionalized with poly(2-(tert-butylamino)ethyl methacrylate) using the grafting technique called SI-ATRP. The results showed that the removal efficiency remained in a fairly wide pH range of pH = 3 to pH = 9, but decreased as pH increased, with maximum adsorption of 290 mg/g. The authors stated that uptake occurs through multiple mechanisms, considering the variation depending on pH. There are likely electrostatic and π-π interactions [200]. 

Plohl et al., on the other hand, focused on the precision delivery of organophosphorus pesticides in order to mitigate their potential negative impact on the environment. For this aim, they utilized nanosystems synthesized to have highly ordered mesoporous channels of around 2.9 nm in size. These nanomaterials were then functionalized with branched PEI (polyethyleneimine) and used for the delivery of the organophosphorus pesticide used as a model, called citridiol, which release kinetics were analysed. The results showed a rapid initial release that stabilized over time, reaching equilibrium after about 10 days. The authors suggest that the use of nanostructured carriers could bring benefits in the use of these types of substances mitigating the risks to human health and the environment [201].

The applications of polymer-modified silica nanocomposites for environmental purposes go beyond their capacity to adsorb and eliminate pollutants. One example is the targeted delivery of agrochemicals, such as pesticides, which can contribute to environmental protection. This approach can result in reduced use of pesticides, enhanced drug performance, and lower risks of environmental contamination. To achieve this objective, it is crucial to improve the effectiveness of pesticides through smart delivery systems. A temperature-responsive release formulation known as THI@HMS@P(NIPAM-MAA) has been proposed to achieve this goal. This formulation can regulate the release of pesticides according to the ambient temperature, thereby optimizing the efficacy of the pesticide. The THI@HMS@P(NIPAM-MAA) was developed using a seeded precipitation polymerization technique, with hollow mesoporous silica (HMS) as the core, a thermoresponsive copolymer named poly(N-isopropylacrylamide-co-methacrylic acid) (P(NIPAM-MAA)) as the outer shell, and thiamethoxam (THI) as the model pesticide. The release of THI was found to increase with temperature, exploiting the correlation between pesticide efficacy and environmental temperature. Additionally, the THI@HMS@P(NIPAM-MAA) exhibited biocompatibility, UV-shielding properties, and strong adhesive properties on rice leaves, resulting in a longer protection period than the naked THI formulation. Thus, the developed temperature-responsive formulation holds great potential as a pest management strategy that can improve pesticide effectiveness and minimize usage [202].

The adsorption for analytical purposes of certain substances can be useful for environmental preservation Xu et al. studied the use of Mesoporous Molecularly Imprinted core-shell (MIP-core shell) silica nanoparticles for the detection and adsorption of atrazine. The functionalization of the nanoparticles was carried out through a technique called reversible addition-fragmentation chain transfer (RAFT) which allowed the preparation of monodisperse, spherical and polymer-coated nanoparticles. The authors demonstrated how the use of this simple and effective method of preparation of MIP-Silica nanoparticles characterized by useful features such as uniform morphology, controllable layer thickness and the possibility of introducing additional vinyl groups, is effective if applied to the adsorption of atrazine not for remediation purposes but for analytical purposes [203].

The same was performed by Song et al., who developed a new method to efficiently extract aflatoxin from cereals. Aflatoxins (AF) are produced by Aspergillus flavus and Aspergillus parasitica and are toxic metabolites with serious health effects, including carcinogenicity. AFs are widespread in crops worldwide, including peanuts, maize, soya, and wheat, and over 20 different types of AFs have been identified, of which AFG2, AFG1, AFB2 and AFB1 are the most harmful to humans. To combat this threat to food safety and human health, researchers have developed new methods to detect AFs. The authors use a mesoporous silica surface-imprinted polymer prepared using chemical grafting rather than the traditional physical wrapping of the carrier material with the imprinting layer. The researchers made the MIP starting from the functional monomer α-methacrylic acid and the crosslinker ethylene glycol dimethacrylate, both based on the C=C bond. The resulting polymerisation layer was more controllable and resulted in a new composite material with specific selectivity, high adsorption capacity and excellent stability. This imprinted polymer was used as solid phase extraction (SPE) column packing used for the separation and enrichment of AF in grain samples [204]. 

Also, worth mentioning, is the work of Shao et al. on the adsorption of Tetrabromobisphenol A (TBBPA) using TiO_2_-coated magnetite-silica nanoparticles functionalised with MIP. The MIP was prepared using the RAFT technique—(reversible addition-fragmentation chain transfer). According to the results of the study, the obtained material shows excellent adsorptive properties towards TBBPA. In addition, the magnetic nature also allows rapid recovery of the adsorbent through the application of a magnetic field and subsequent reuse for at least five cycles without a significant loss of adsorption efficiency [205].

## 4. Conclusions

The ability of polymer-silica nanostructured materials to remove organic and inorganic pollutants from water and air has been extensively studied and reviewed. Polymer-silica nanostructured materials can effectively remove organic pollutants and heavy metals from water and air due to their high distribution of surface functional groups and specific physico-chemical characteristics. Several studies have demonstrated the efficacy of these applications making them a promising alternative to conventional methods. Despite polymer-silica, nanostructured materials show excellent potential for environmental remediation; however, there are still challenges that need to be addressed. One of the major challenges is the matter of the mechanical properties of the materials. Polymer-silica nanostructured materials can be fragile and prone to mechanical failure, limiting their durability and reusability. Further extensive research is necessary to improve the mechanical properties of these materials, as well as their applicability for repeated cycles. Furthermore, it is important to take into account the necessity of developing a binder that provides the nanostructured materials with the desired rigidity of a fixed bed, while simultaneously preserving as much as possible their inherent characteristics of high pore volumes and specific surface area. 

Another challenge is related to the functionalization of the materials, as it plays a crucial role in obtaining nanoadsorbent for specific applications: it can be challenging to achieve uniform functionalization of the materials potentially affecting their performance. Despite the very large number of proposed applications, it is important to acknowledge that each one could be far from being the optimal solution. However, we can surely affirm that materials engineering is emerging as the only approach for developing the most promising candidates, the trend is evident in recent publications, to improve the sustainability of environmental issues. 

## Figures and Tables

**Table 1:** Polymer-based nanomaterial and applications in heavy metal adsorption.

Silica	Polymer	Elements Retained by the Silica/Polymer Nanocomposite	Ref.
MCM-41	PAA (Polyacrylamide-co-acrylic acid)	Pb (II)/Cd (II)	[110]
MCM-41	PVA (Poly(vinyl alcohol))	Cd (II)	[111]
Fe_3_O_4__Silica	PmAP (Poly(m-aminophenol))	Hg (II)	[113]
MCM-41	PMMA (Poly-methyl methacrylate)	Cu (II)	[104]
γ-Fe_2_O_3__Silica	CMC (Amino-biopolymer carboxymethyl chitosan)	Cu (II)	[105]
Silica	Chitosan + PMMA (Poly(methyl methacrylate)	Cr (VI)	[121]
SBA-15	PVP (Polyvinylpyrrolidone)	Pb (II)/Ni (II)/Cd (II)	[107]
Fe_3_O_4__SBA-15	Imprinted Polymer using Methacrylic Acid	Pb (II)/Cd (II)	[109]
Silica	COL (Chitosan oligosaccharide lactate)	Hg (II)	[103]
MCM-48	β-CD (β-cyclodextrin)	As (V)/Hg (II)	[112]
Silica	PANAM (Polyacrylonitrile-acrylamide)	Pb (II)/Cs (II)/Cu (II)/Cd (II)/Sr (II)	[106]
Silica	Copolymerized 1-vinyl-imidazole + acrylic acid	Ni (II)	[108]
Silica-coated magnetic nanoparticles (MMS)	Amidoxime (MMS-AO)	U (VI)	[115]
MCM-41	PAN (Polyacrylonitrile) + Amidoxime	U (VI)	[116]
MMSNs	PPI (Poly(propyleneimine) dendrimer)) and PANAM (Polyamidoamine dendrimer)	U (VI)	[117]
MMSNs	PPI (Poly(propyleneimine) dendrimer)) and PANAM (Polyamidoamine dendrimer)	U (VI)	[118]
MCM-48	Chitosan	U (VI)/Sr (II)	[119]
DFNS (Fibrous dendritic silica)	PANAM (Poly(amidoamine))	U (VI)	[120]

**Table 2:** Performance of polymer-based nanomaterial in heavy metal adsorption.

Nanocomposite	Adsorbate	Removal Rate	Adsorption Capacity	Recycling	Ref.
MCM-41-PMMA (Poly-methyl methacrylate)	Cu (II)	------	40.95 mg/g	------	[104]
γ- Fe_2_O_3__Silica-CMC (Amino-biopolymer carboxymethyl chitosan)	Cu (II)	35%	≈ 350 mg/g	4 cycles	[105]
SBA-15–PVP (Polyvinylpyrrolidone)	Pb (II)/Ni (II)/Cu (II)	Pb (II) = 52% Ni (II) = 44% Cu (II) = 35%	Pb (II) = 175 mg/g Ni (II) = 72 mg/g Cu (II) = 128 mg/g	------	[107]
Silica Copolymerized 1-vinyl-imidazole + acrylic acid	Ni (II)	99.40%	62.81 mg/g	5 cycles	[108]
Fe_3_O_4__SBA-15–Imprinted Polymer using Methacrylic Acid	Pb (II)/Cd (II)	Pb (II) = 90.11% Cd (II) = 94.55%	Pb (II) = 18.18 mg/g Cd (II) = 14.28 mg/g	5 cycles	[109]
MCM-41 PAA (Polyacrylamide-co-acrylic acid)	Pb (II)/Cd (II)	Pb (II) = 60–83% Cd (II) = 88–98%	Pb (II) = 14.77 mg/g Cd (II) = 21.15 mg/g	10 cycles	[110]
MCM-41–PVA (Poly(vinyl alcohol))	Cd (II)	------	46.73 mg/g	------	[111]
Silica-COL (Chitosan oligosaccharide lactate)	Hg (II)	77.98%	116.7 mg/g	------	[103]
MCM-48–β-CD (β-cyclodextrin)	As (V)/Hg (II)	As (V) = 72.8% Hg (II) = 60.4%	As (V) = 265.6 mg/g Hg (II) = 207.9 mg/g	------	[112]
Fe_3_O_4__Silica–PmAP (Poly(m-aminothiophenol)	Hg (II)	97.53%	243.98 mg/g	5 cycles	[113]
Mesoporous silica-coated magnetic nanoparticles (MMS)–Amidoxime (MMS-AO)	U (VI)	> 99%	277.3 mg/g	5 cycles	[115]
MCM-41–PAN (Polyacrylonitrile + Amidoxime)	U (VI)	97%	442.3 mg/g	10 cycles	[116]
MMSNs–PPI (Polypropylene imine dendrimer) and PAMAM (Polyamidoamine dendrimer)	U (VI)	------	PPI = 133.3 mg/g PAMAM = 53.8 mg/g	5 cycles	[118]
MCM-48—Chitosan	U (VI)/Sr (II)	U (VI) = 100% at 1.2 g/L of adsorbent Sr (II) = 100% at 1.2 g/L of adsorbent	U (VI) = 260 mg/g Sr (II) = 330 mg/g	5 cycles	[119]
DFNS (Fibrous dendritic silica)–PANAM (Polyamidoamine)	U (VI)	>90%	215.2 mg/g	5 cycles	[120]
Silica–Chitosan + PMMA (Polymethyl methacrylate)	Cr (VI)	98%	92.5 mg/L	------	[121]

**Table 3:** Polymer-based nanomaterial for applications in dye adsorption.

Material	Polymer	Adsorbate	Ref.
Vinyl Modified-Silica-Magnetic-Nanoparticles	Acrylic Acid (AA) 2-acrylamido-2-methyl-1-propanesulfonic acid (AMPS)	Methylen Blue 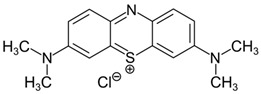	[134]
MCM-41	Polyglycerolmethacrylate poly(GDMA)	Methylen Blue	[139]
KIT-6	β-cyclodextrin	Methylen Blue	[142]
Silica Nanoparticles	Acrylic acrylamide (SAA)	Methylen Blue	[144]
SBA-15	Polyvinyl alcohol (PVA)	Methylen Blue	[145]
Silica Nanoparticles	Poly(N-isopropylacrylamide)	Methylen Blue	[146]
SBA-15	Polypyrrole	Methylen Blue Methyl Orange 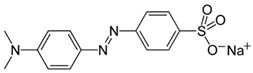	[147]
Fe_3_O_4_@MSN	Poly(2-diethylaminoethyl methacrylate) (PDEAEMA)	Methyl Orange E110—Sunset Yellow (SY) 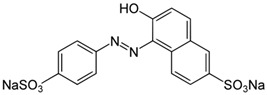	[151]
Magnetic silica nanoparticles	Poly(2-methacryloyloxy)ethyl trimethylammonium chloride—(PMETAC)	Methyl Orange Bromothymol Blue 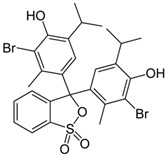	[152]
SBA-15	Poly(amidoamine)	Acid Blue 62 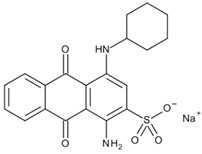	[153]
MCM—41	Polypirrole–PPy Polyaniline–PAni	Acid Blue 62	[154]
Silica magnetic nanoparticles-Aptes modified	Molecular Imprinted Polymer	Congo Red 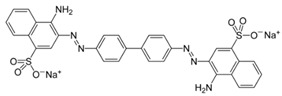	[155]
SBA-15	Cross-linked poly-(acrylic acid) (CPAA)	Acid Blue 25 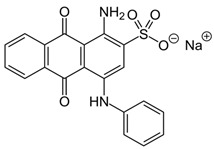	[156]
Magnetic MCM-41	Poly(2-methacryloyloxy)ethyl] trimethylammonium chloride—(PMETAC)	Direct Blue 6 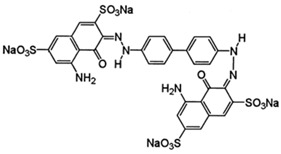 Direct Black 38 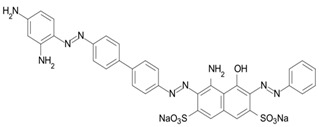	[158]
Magnetic Silica Nanoparticles	Zwitterionic polymer derived from: poly(2-(tert-butylamino)ethyl methacrylate)	Rhodamine B (Rh B) 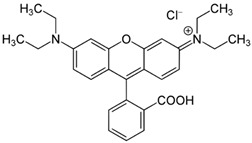 Crystal Violet (CV) 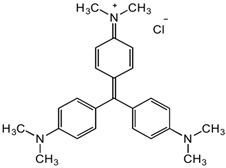	[85]
MSN (Mesoporous Silica Nanoparticles)	Hyper-Crosslinked Polymer, based on vinylbenzyl chloride and divinylbenzene	Remazol Brilliant Blue R 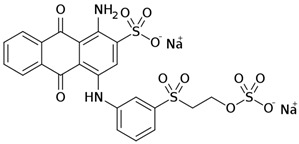	[159]
SBA-15	Chitosan	Brilliant Red E-B4A 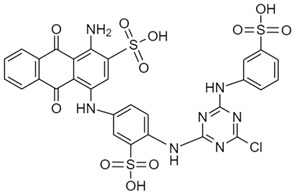	[160]
SBA-15	Polyaniline	Reactive Orange 16 (RO 16) 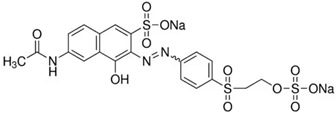	[161]

**Table 4:** Polymer-based nanomaterial and their applications in drug adsorption.

Material	Polymer	Adsorbate	Ref.
Mesoporous silica nanoparticles	Poly(2-(tert-butylamino)ethyl methacrylate) (PTBAEMA)	Paracetamol 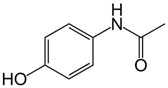	[170]
SBA-15	Polypirrole based molecularly imprinted polymers	Acido Ascorbico 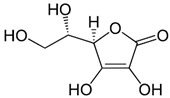	[168]
Stober Silica Nanoparticles	Molecularly imprinted polymers	17β-Estradiol 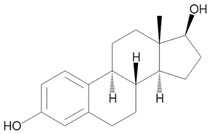	[169]
SBA-15	Molecularly imprinted polymers	Salicylic acid 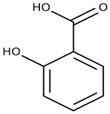	[167]
Core–shell aminografted silica nanoparticle	Polyvinyl alcohol chitosan	Diclofenac sodium 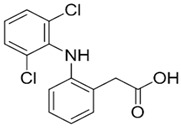	[171]

## Data Availability

No data was used for the research described in the article.
